# Supplementary material for: Knockdown of hsa_circ_0008922 inhibits the progression of glioma
Source: PeerJ. 2022 Dec 20;10:e14552. doi: 10.7717/peerj.14552 (PMC9784332; doi:10.7717/peerj.14552)
Supplement: Supplemental Information 1 — Raw data for Figure 9. [file peerj-10-14552-s001.docx]

Additional Table 1 The enriched top 30 GO terms

| GO_id | GO_name | GO_diffgene_count | Enrichment | *P* |
| --- | --- | --- | --- | --- |
| GO:0007064 | mitotic sister chromatid cohesion | 2 | 60.46259 | 0.000469 |
| GO:0050771 | negative regulation of axonogenesis | 2 | 41.85871 | 0.001006 |
| GO:0001205 | transcriptional activator activity, RNA polymerase II distal enhancer sequence-specific DNA binding | 2 | 25.81458 | 0.00267 |
| GO:0006355 | regulation of transcription, DNA-templated | 10 | 2.675336 | 0.003392 |
| GO:0019827 | stem cell population maintenance | 2 | 13.9529 | 0.008992 |
| GO:0005524 | ATP binding | 10 | 2.238906 | 0.011584 |
| GO:0045177 | apical part of cell | 2 | 10.61214 | 0.015226 |
| GO:0060261 | positive regulation of transcription initiation from RNA polymerase II promoter | 1 | 54.41633 | 0.018245 |
| GO:0035356 | cellular triglyceride homeostasis | 1 | 54.41633 | 0.018245 |
| GO:0097237 | cellular response to toxic substance | 1 | 54.41633 | 0.018245 |
| GO:0071732 | cellular response to nitric oxide | 1 | 54.41633 | 0.018245 |
| GO:1902166 | negative regulation of intrinsic apoptotic signaling pathway in response to DNA damage by p53 class mediator | 1 | 54.41633 | 0.018245 |
| GO:0010917 | negative regulation of mitochondrial membrane potential | 1 | 54.41633 | 0.018245 |
| GO:0070695 | FHF complex | 1 | 53.06071 | 0.018707 |
| GO:0033503 | HULC complex | 1 | 53.06071 | 0.018707 |
| GO:1990904 | ribonucleoprotein complex | 1 | 53.06071 | 0.018707 |
| GO:0008375 | acetylglucosaminyltransferase activity | 1 | 51.62917 | 0.019222 |
| GO:0003682 | chromatin binding | 4 | 3.881892 | 0.019291 |
| GO:0000776 | kinetochore | 2 | 8.993341 | 0.020813 |

(Continued from the last page)

| GO_id | GO_name | GO_diffgene_count | Enrichment | *P* |
| --- | --- | --- | --- | --- |
| GO:0016567 | protein ubiquitination | 4 | 3.765836 | 0.021345 |
| GO:0043951 | negative regulation of cAMP-mediated signaling | 1 | 45.34694 | 0.021855 |
| GO:0030240 | skeletal muscle thin filament assembly | 1 | 45.34694 | 0.021855 |
| GO:0033523 | histone H2B ubiquitination | 1 | 45.34694 | 0.021855 |
| GO:1990416 | cellular response to brain-derived neurotrophic factor stimulus | 1 | 45.34693878 | 0.021854631 |
| GO:0031062 | positive regulation of histone methylation | 1 | 45.34693878 | 0.021854631 |
| GO:0046324 | regulation of glucose import | 1 | 45.34693878 | 0.021854631 |
| GO:0000083 | regulation of transcription involved in G1/S transition of mitotic cell cycle | 1 | 45.34693878 | 0.021854631 |
| GO:0031463 | Cul3-RING ubiquitin ligase complex | 2 | 8.698477752 | 0.022154479 |
| GO:0008278 | cohesin complex | 1 | 44.2172619 | 0.022407295 |
| GO:0030893 | meiotic cohesin complex | 1 | 44.2172619 | 0.022407295 |
