# Supplementary material for: Knockdown of hsa_circ_0008922 inhibits the progression of glioma
Source: PeerJ. 2022 Dec 20;10:e14552. doi: 10.7717/peerj.14552 (PMC9784332; doi:10.7717/peerj.14552)
Supplement: Supplemental Information 2 — Raw data for Figure 9. [file peerj-10-14552-s002.docx]

Additional Table 2 The enriched top 30 KEGG pathways

| Path_id | Path_name | Path_diffgene_count | Enrichment | *P* |
| --- | --- | --- | --- | --- |
| path:hsa04550 | Signaling pathways regulating pluripotency of stem cells | 4 | 6.726386633 | 0.00272897 |
| path:hsa04110 | Cell cycle | 3 | 5.655046826 | 0.01550824 |
| path:hsa05206 | MicroRNAs in cancer | 3 | 4.67483871 | 0.025589244 |
| path:hsa05214 | Glioma | 2 | 6.584279873 | 0.036663929 |
| path:hsa05218 | Melanoma | 2 | 6.492831541 | 0.037612503 |
| path:hsa00061 | Fatty acid biosynthesis | 1 | 17.98014888 | 0.054255194 |
| path:hsa03450 | Non-homologous end-joining | 1 | 17.98014888 | 0.054255194 |
| path:hsa01522 | Endocrine resistance | 2 | 4.869623656 | 0.063013869 |
| path:hsa05215 | Prostate cancer | 2 | 4.81942135 | 0.064172732 |
| path:hsa04914 | Progesterone-mediated oocyte maturation | 2 | 4.722059303 | 0.066512342 |
| path:hsa00240 | Pyrimidine metabolism | 2 | 4.628553178 | 0.068880609 |
| path:hsa04014 | Ras signaling pathway | 3 | 3.022525028 | 0.075503042 |
| path:hsa00670 | One carbon pool by folate | 1 | 11.68709677 | 0.082278096 |
| path:hsa00532 | Glycosaminoglycan biosynthesis - chondroitin sulfate / dermatan sulfate | 1 | 11.68709677 | 0.082278096 |
| path:hsa04152 | AMPK signaling pathway | 2 | 3.895698925 | 0.092692394 |
| path:hsa00534 | Glycosaminoglycan biosynthesis - heparan sulfate / heparin | 1 | 9.739247312 | 0.097928613 |
| path:hsa04114 | Oocyte meiosis | 2 | 3.770031217 | 0.097980382 |
| path:hsa05310 | Asthma | 1 | 8.347926267 | 0.113320699 |
| path:hsa00630 | Glyoxylate and dicarboxylate metabolism | 1 | 7.791397849 | 0.120921122 |
| path:hsa03020 | RNA polymerase | 1 | 7.540062435 | 0.124697653 |
| path:hsa01523 | Antifolate resistance | 1 | 7.540062435 | 0.124697653 |
| path:hsa04060 | Cytokine-cytokine receptor interaction | 3 | 2.401458241 | 0.127451225 |
| path:hsa05224 | Breast cancer | 2 | 3.180162388 | 0.129922725 |

(Continued from the last page)

| Path_id | Path_name | Path_diffgene_count | Enrichment | *P* |
| --- | --- | --- | --- | --- |
| path:hsa03410 | Base excision repair | 1 | 7.083088954 | 0.132203669 |
| path:hsa04150 | mTOR signaling pathway | 2 | 3.095919675 | 0.135710639 |
| path:hsa04218 | Cellular senescence | 2 | 2.958758677 | 0.145980121 |
| path:hsa04630 | Jak-STAT signaling pathway | 2 | 2.885702907 | 0.151922785 |
| path:hsa05225 | Hepatocellular carcinoma | 2 | 2.833235582 | 0.156412707 |
| path:hsa00260 | Glycine, serine and threonine metabolism | 1 | 5.843548387 | 0.157987325 |
| path:hsa04216 | Ferroptosis | 1 | 5.843548387 | 0.157987325 |
