# Supplementary material for: Knockdown of hsa_circ_0008922 inhibits the progression of glioma
Source: PeerJ. 2022 Dec 20;10:e14552. doi: 10.7717/peerj.14552 (PMC9784332; doi:10.7717/peerj.14552)
Supplement: Supplemental Information 3 — Raw Pairs: Number of fragments generated from raw sequencing data. Trimmed: Number of fragments (read pairs) resulting from removing 5′ and 3′ splice sequences and filtering out excessively short fragments ≤20 bp. Mapped: reads are mapped to the reference genome. mtRNAs: Ratio of the number of fragments aligned to the mitochondrial genome to the number of Pairs. rRNAs: Ratio of the number of fragments matched to the rRNA to the number of Pairs. Unmapped: reads are not mapped into the reference genome. [file peerj-10-14552-s003.docx]

| Samples | Raw Pairs | Trimmed | mtRNAs | rRNAs | Mapped | Unmapped |
| --- | --- | --- | --- | --- | --- | --- |
| 1 | 31879989 | 31838749 | 3.78% | 3.78% | 89.82% | 10.18% |
| 2 | 35771943 | 35716258 | 2.29% | 0.00% | 90.23% | 9.77% |
| 3 | 28150858 | 28120369 | 2.79% | 0.00% | 88.34% | 11.66% |
| 4 | 32692605 | 32659018 | 5.90% | 0.00% | 91.42% | 8.58% |
| 5 | 31444657 | 31400649 | 6.21% | 0.00% | 91.58% | 8.42% |
| 6 | 31966476 | 31932390 | 7.20% | 0.00% | 91.58% | 8.42% |
| 7 | 35578325 | 35554281 | 3.52% | 0.00% | 88.01% | 11.99% |
| 8 | 32635163 | 32573974 | 3.40% | 0.00% | 90.80% | 9.20% |
| 9 | 29397538 | 29351715 | 2.63% | 0.00% | 89.11% | 10.89% |
| 10 | 28263024 | 28211325 | 2.72% | 0.00% | 90.57% | 9.43% |

Additional Table 3: Reads comparison result statistics

Raw Pairs: Number of fragments generated from raw sequencing data.

Trimmed: Number of fragments (read pairs) resulting from removing 5' and 3' splice sequences and filtering out excessively short fragments ≤20 bp.

Mapped: reads are mapped to the reference genome.

mtRNAs: Ratio of the number of fragments aligned to the mitochondrial genome to the number of Pairs.

rRNAs: Ratio of the number of fragments matched to the rRNA to the number of Pairs.

Unmapped: reads are not mapped into the reference genome.
