# Supplementary figures and images for: Knockdown of hsa_circ_0008922 inhibits the progression of glioma
Source: PeerJ. 2022 Dec 20;10:e14552. doi: 10.7717/peerj.14552 (PMC9784332; doi:10.7717/peerj.14552)

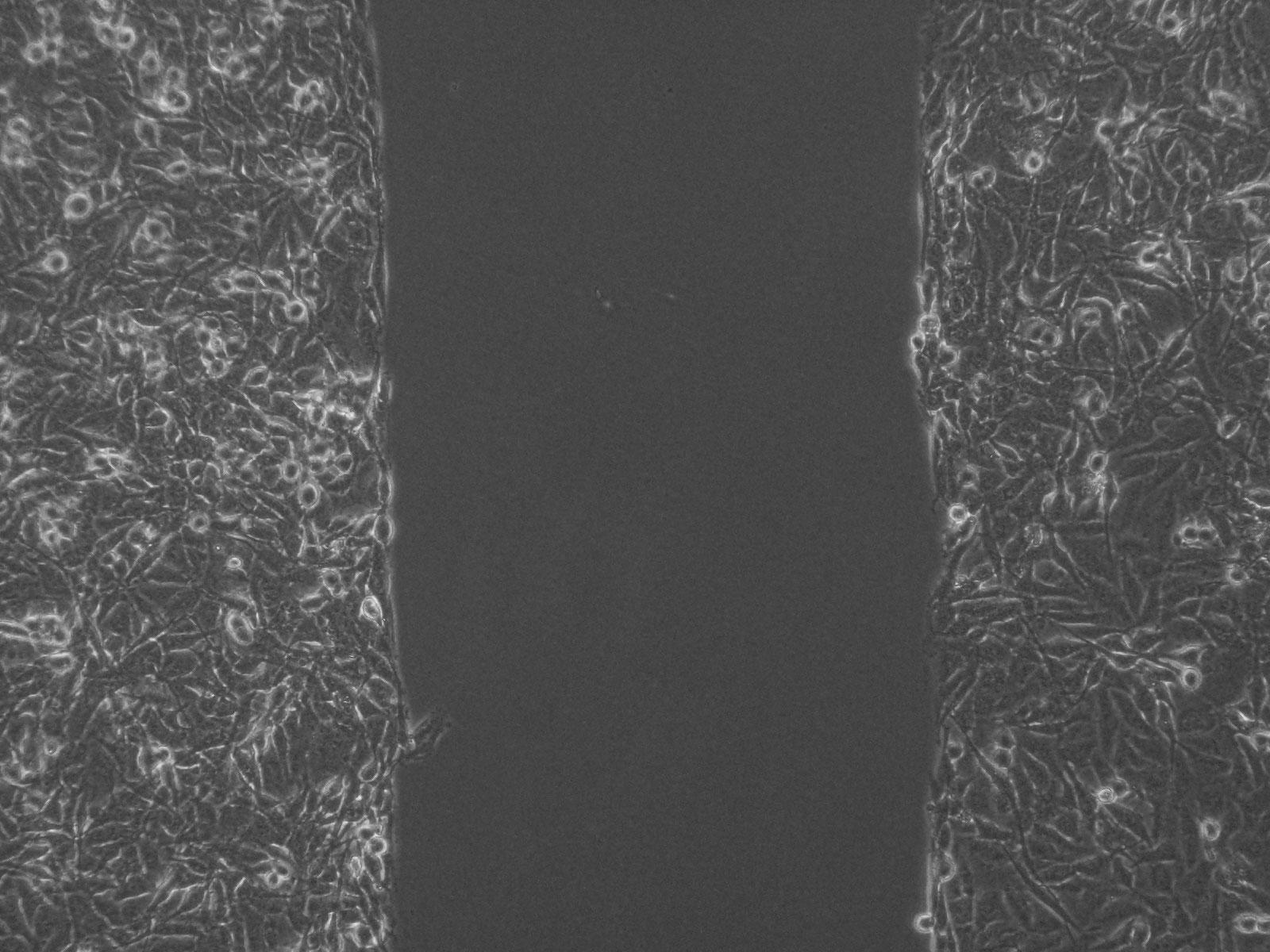

Supplement: Supplemental Information 8 [file peerj-10-14552-s008.zip › 图压缩版/Figure 4/A172-0H-NC.jpg]

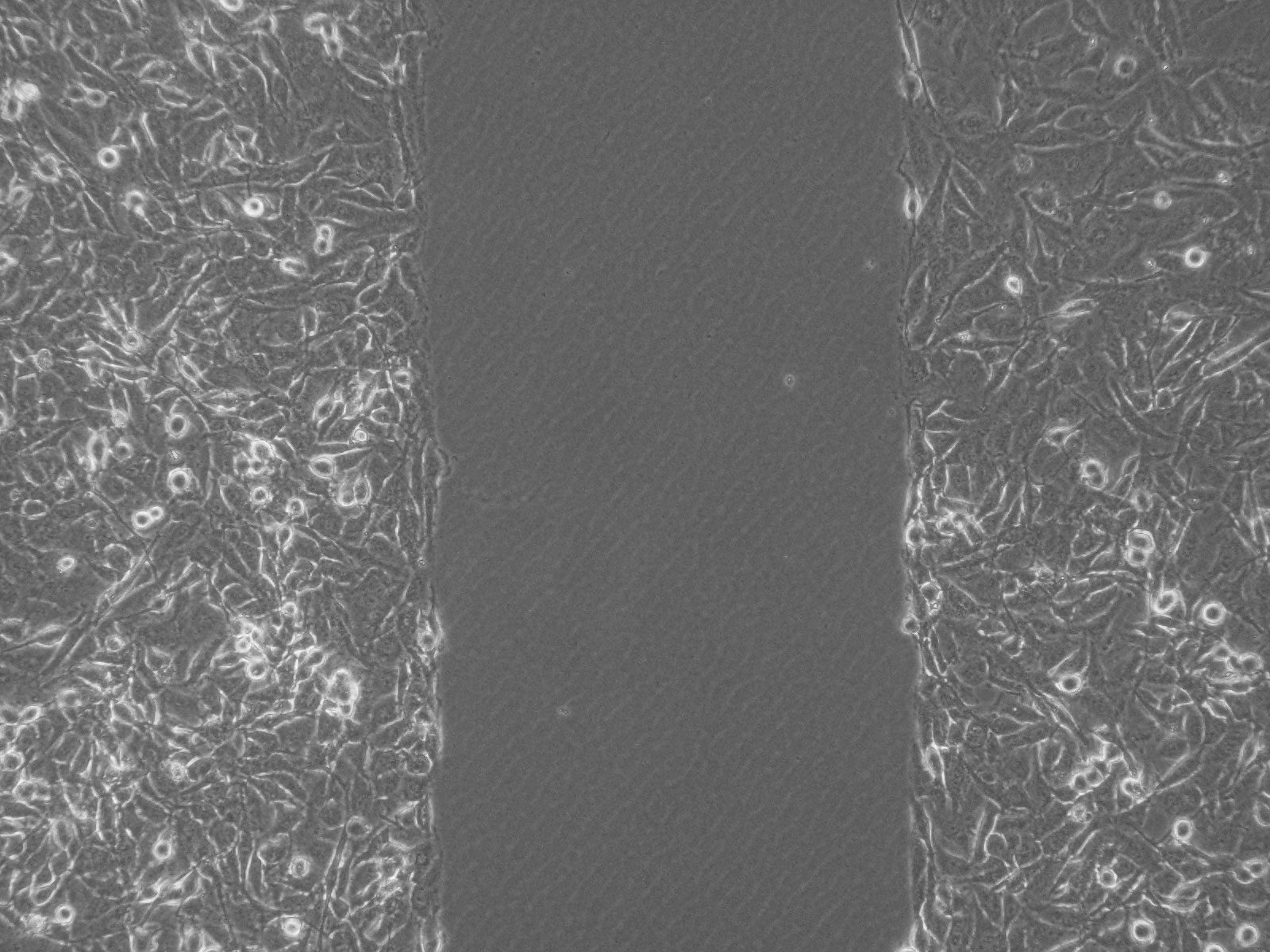

Supplement: Supplemental Information 8 [file peerj-10-14552-s008.zip › 图压缩版/Figure 4/A172-0H-s2-hsa_circ_008922.jpg]

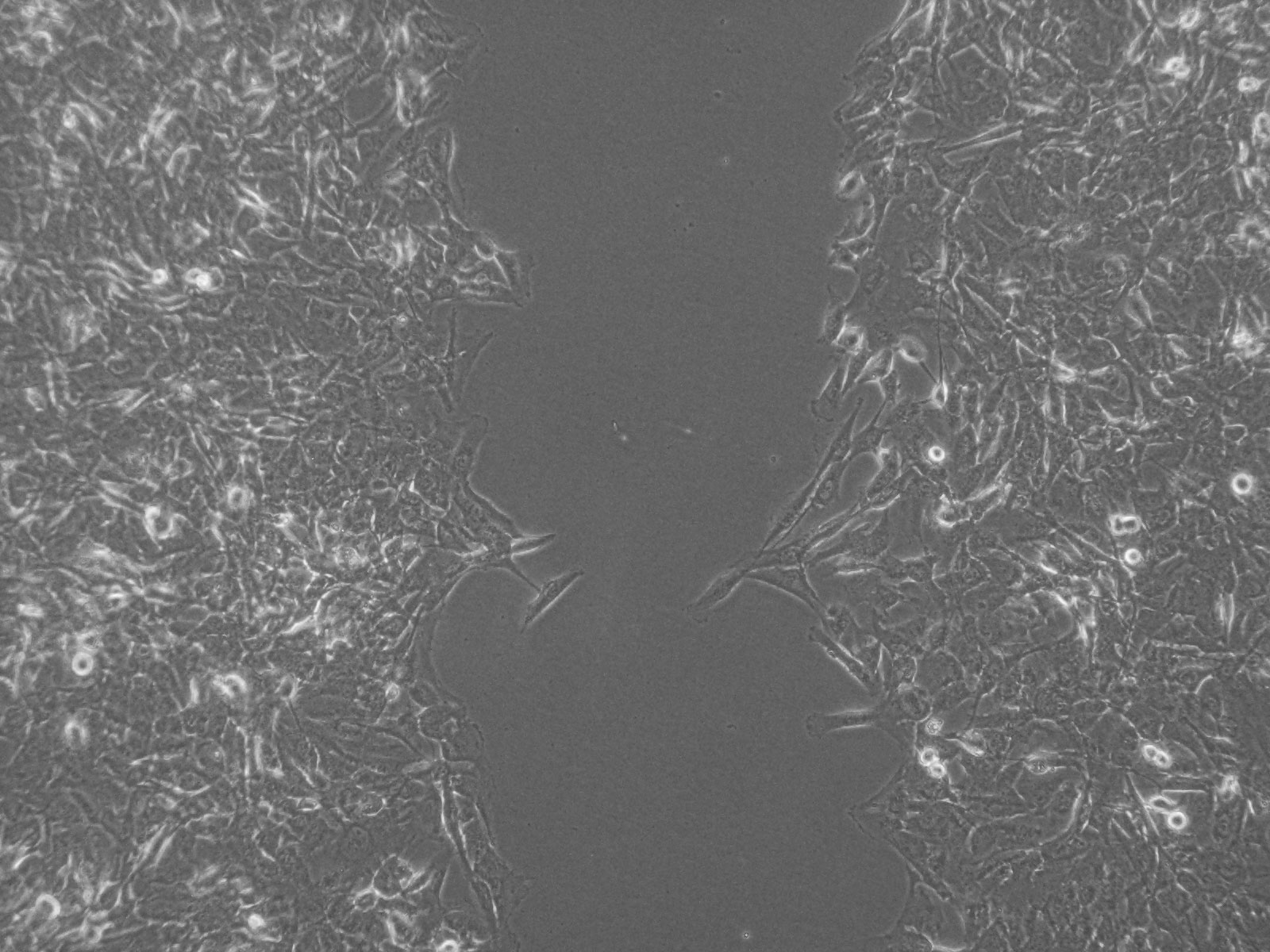

Supplement: Supplemental Information 8 [file peerj-10-14552-s008.zip › 图压缩版/Figure 4/A172-12H-NC.jpg]

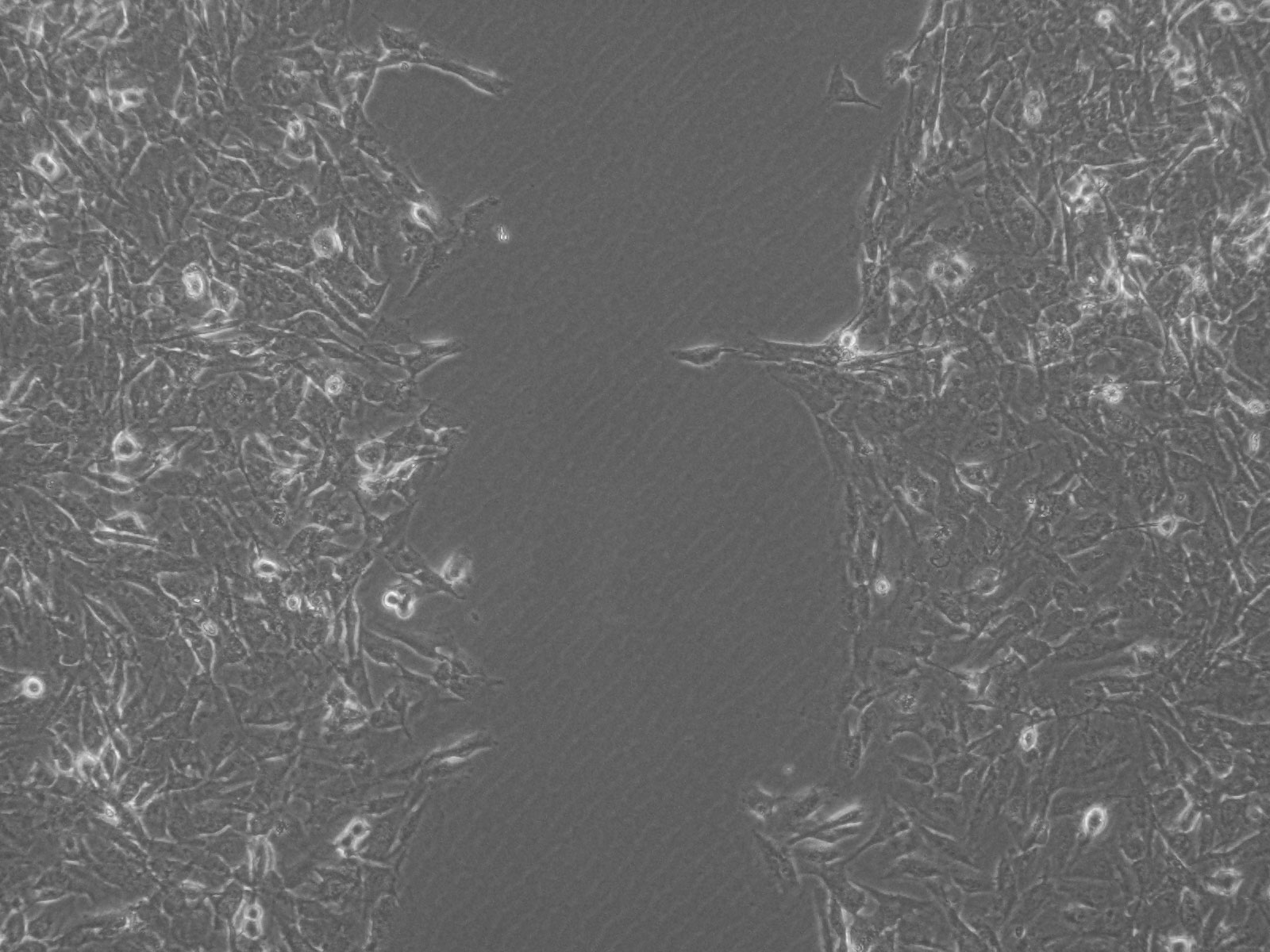

Supplement: Supplemental Information 8 [file peerj-10-14552-s008.zip › 图压缩版/Figure 4/A172-12H-s2-hsa_circ_008922.jpg]

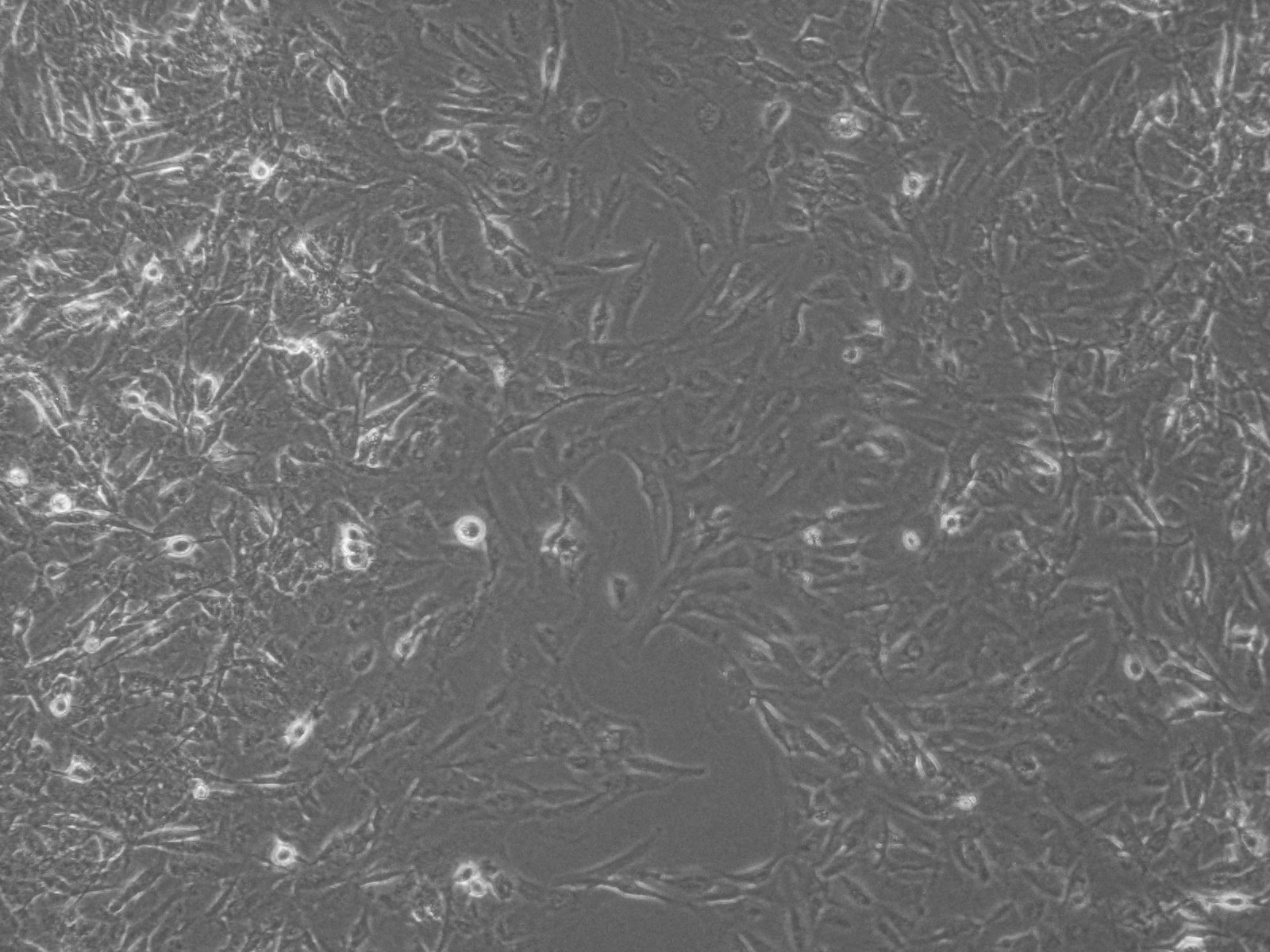

Supplement: Supplemental Information 8 [file peerj-10-14552-s008.zip › 图压缩版/Figure 4/A172-24H-NC.jpg]

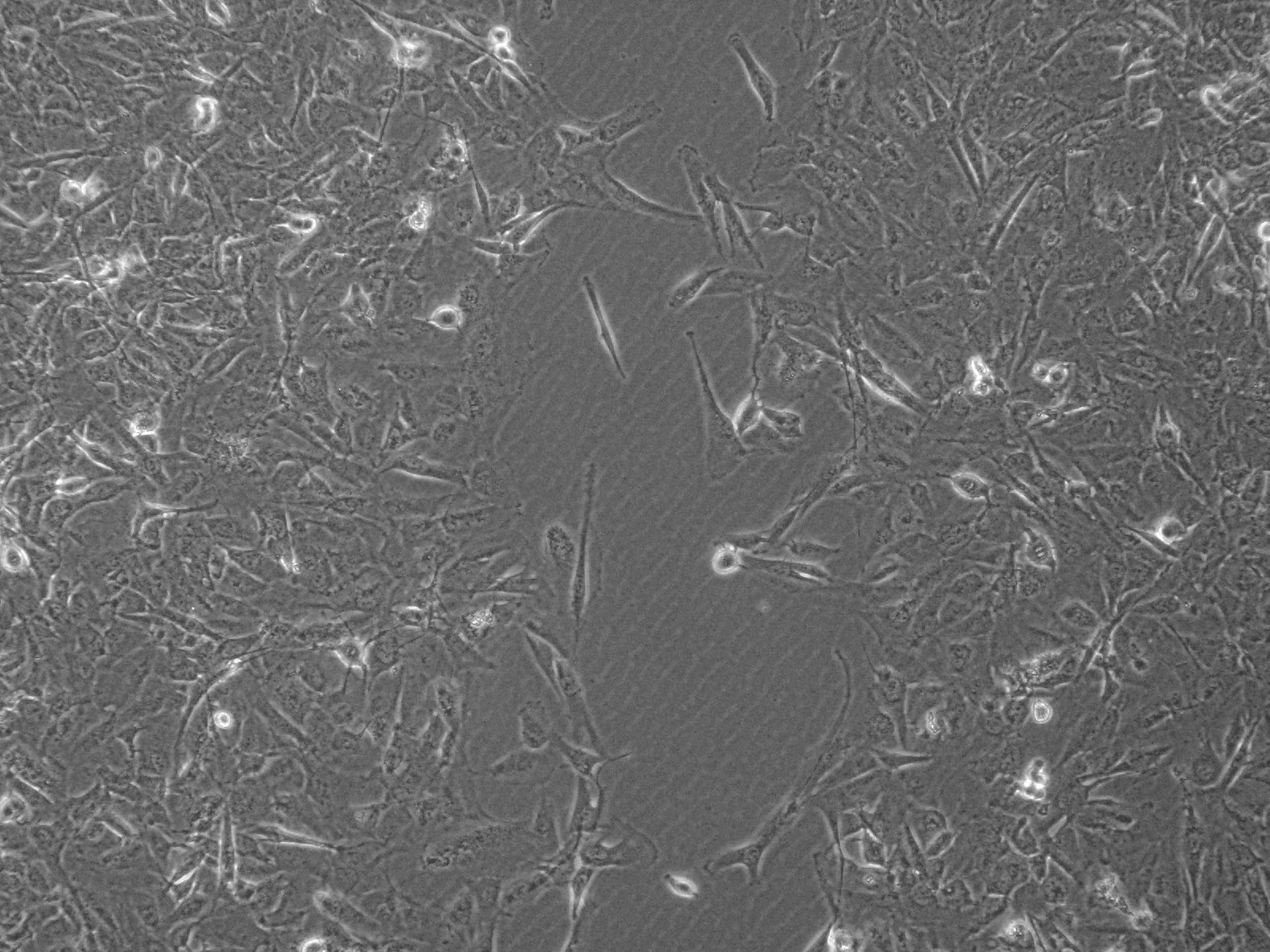

Supplement: Supplemental Information 8 [file peerj-10-14552-s008.zip › 图压缩版/Figure 4/A172-24H-s2-hsa_circ_008922.jpg]

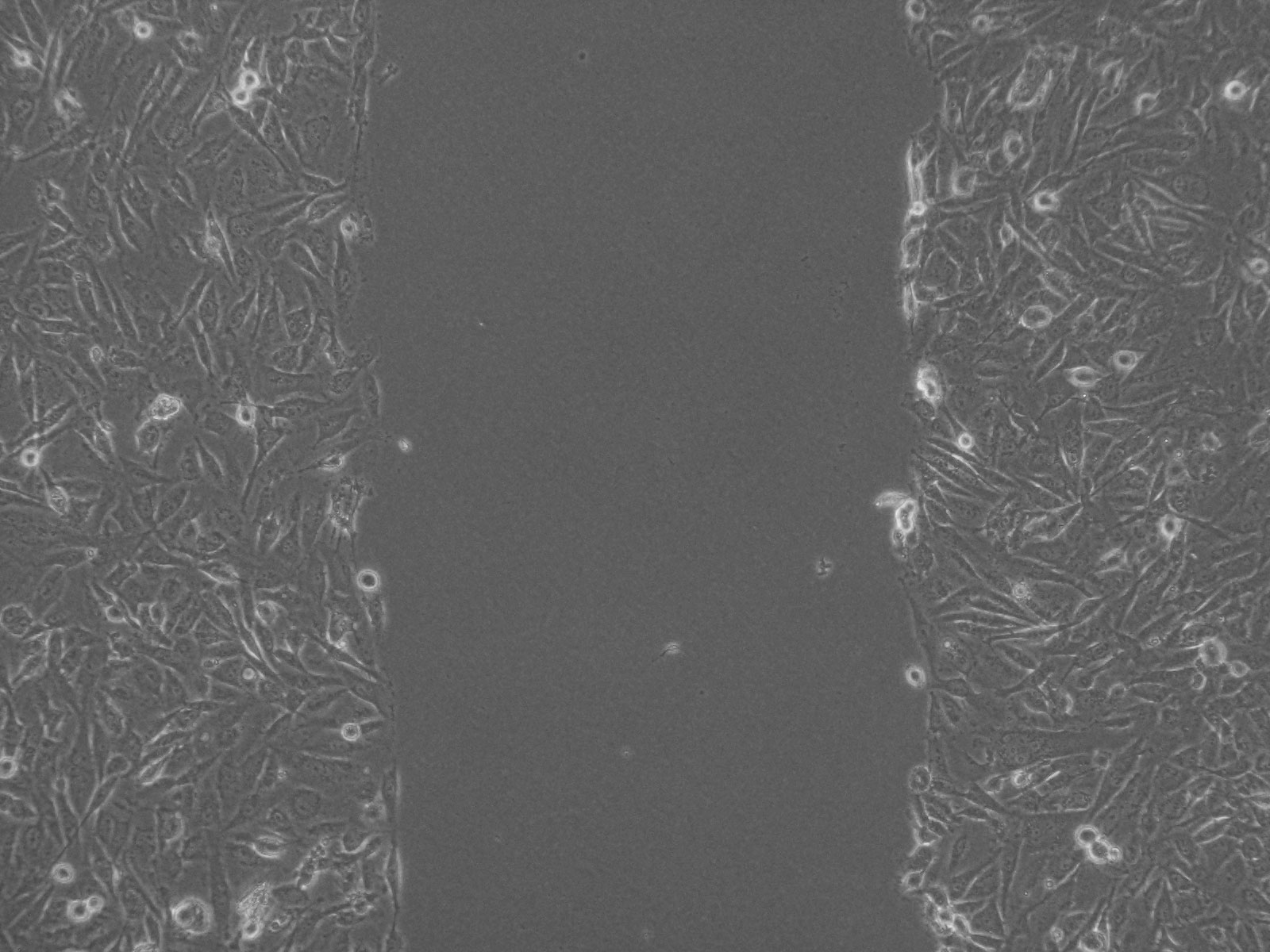

Supplement: Supplemental Information 8 [file peerj-10-14552-s008.zip › 图压缩版/Figure 4/U251-0H-NC.jpg]

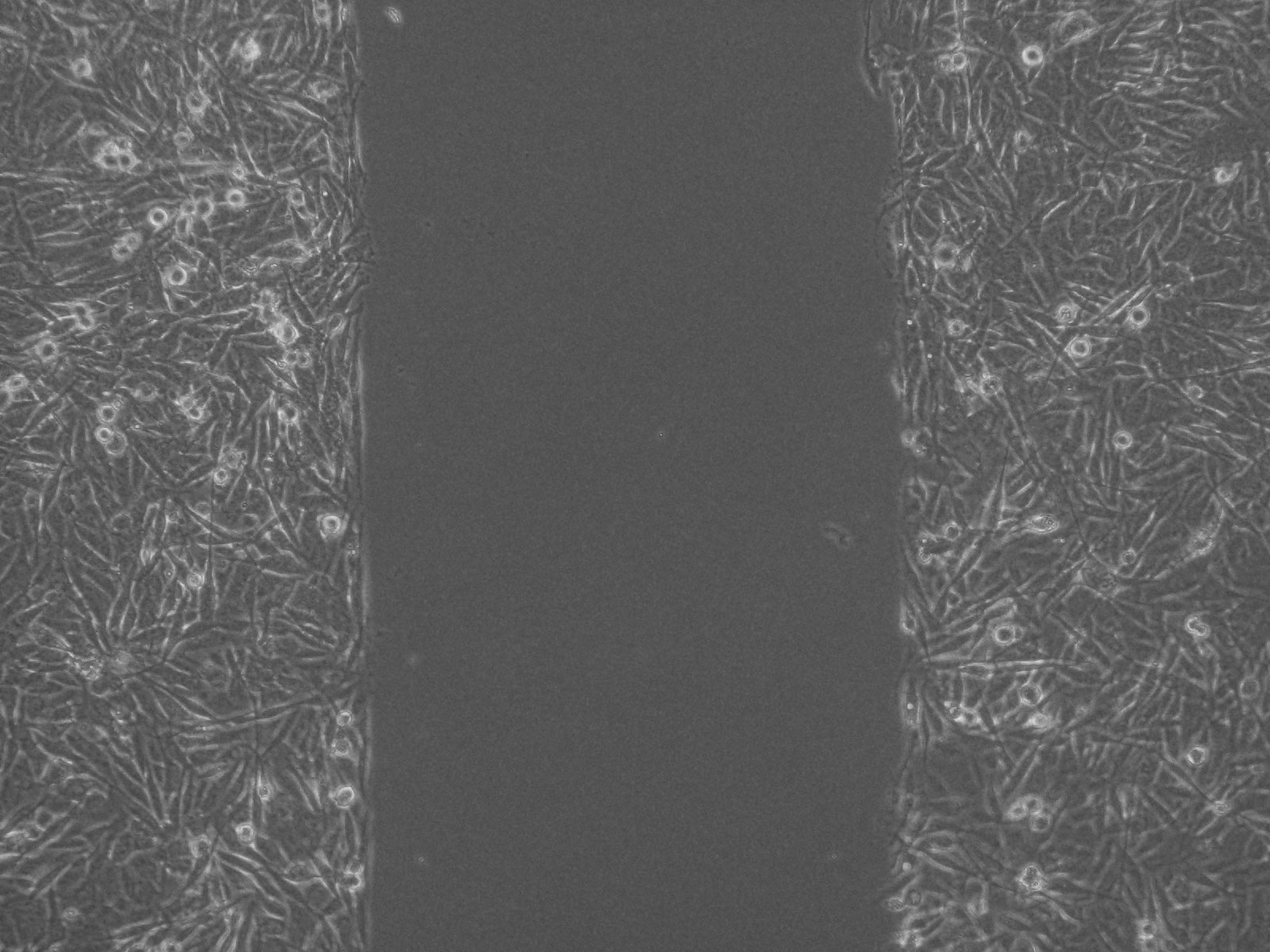

Supplement: Supplemental Information 8 [file peerj-10-14552-s008.zip › 图压缩版/Figure 4/U251-0H-s2-hsa_circ_008922.jpg]

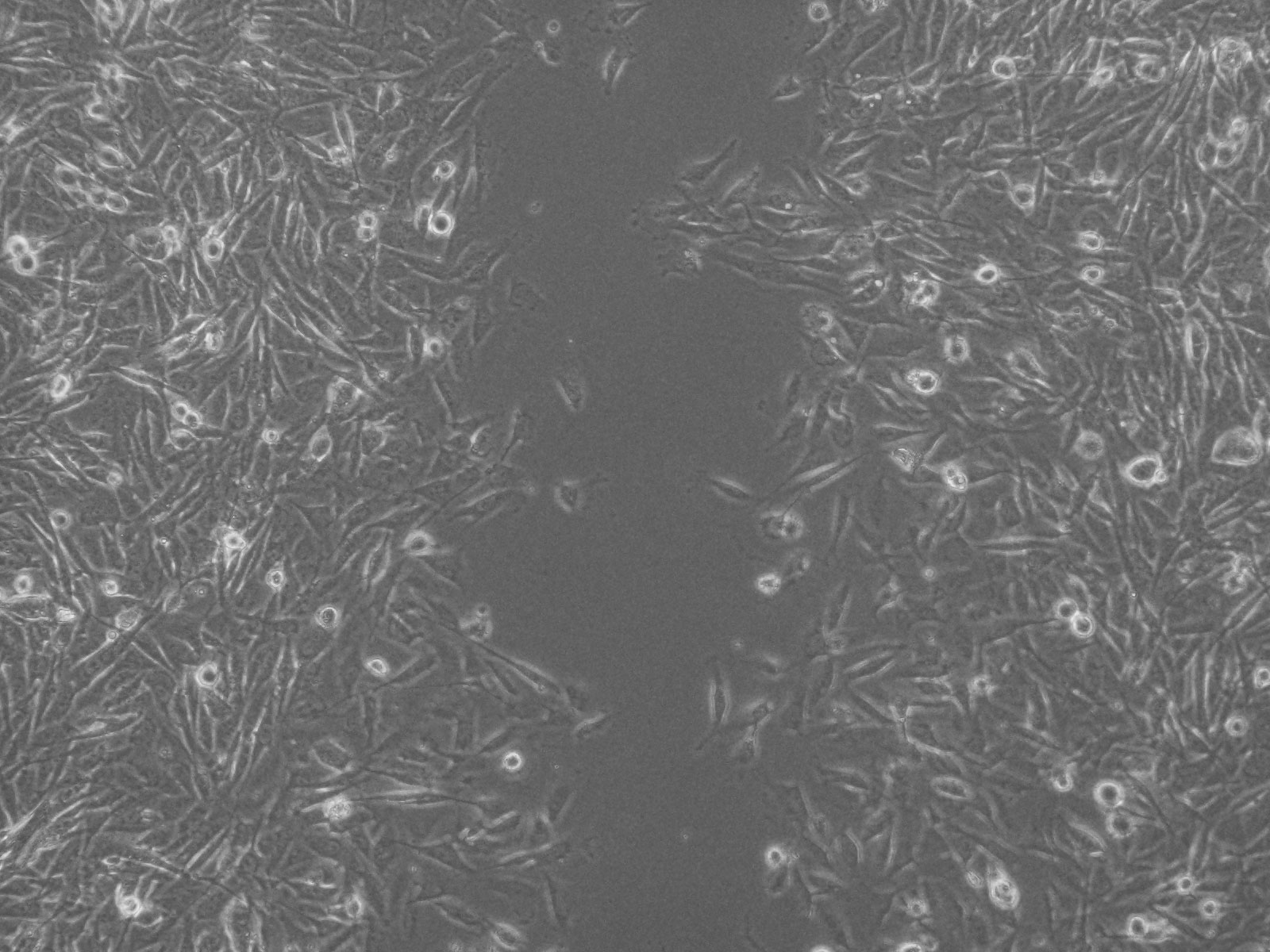

Supplement: Supplemental Information 8 [file peerj-10-14552-s008.zip › 图压缩版/Figure 4/U251-12H-NC.jpg]

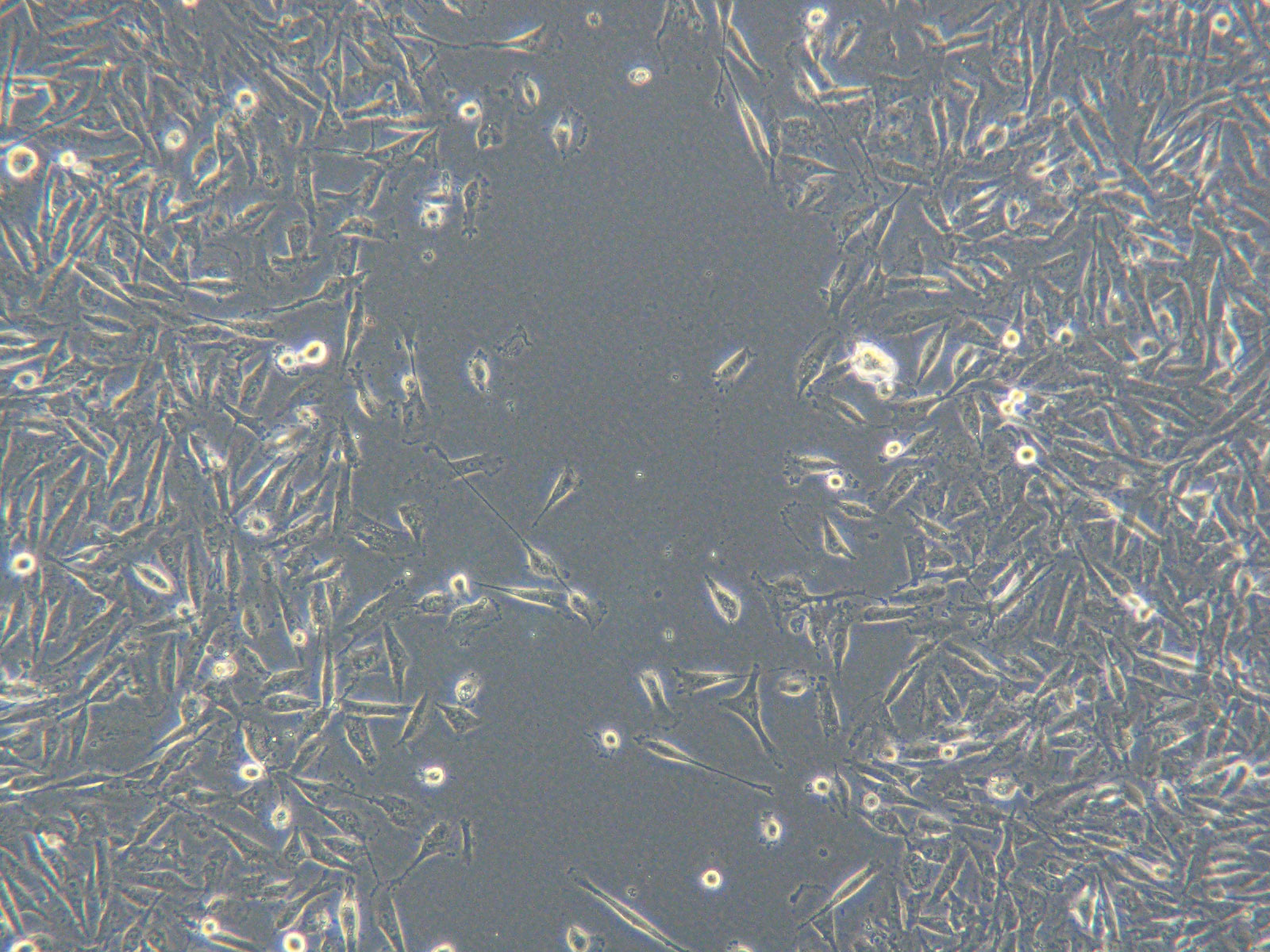

Supplement: Supplemental Information 8 [file peerj-10-14552-s008.zip › 图压缩版/Figure 4/U251-12H-s2-hsa_circ_008922.jpg]

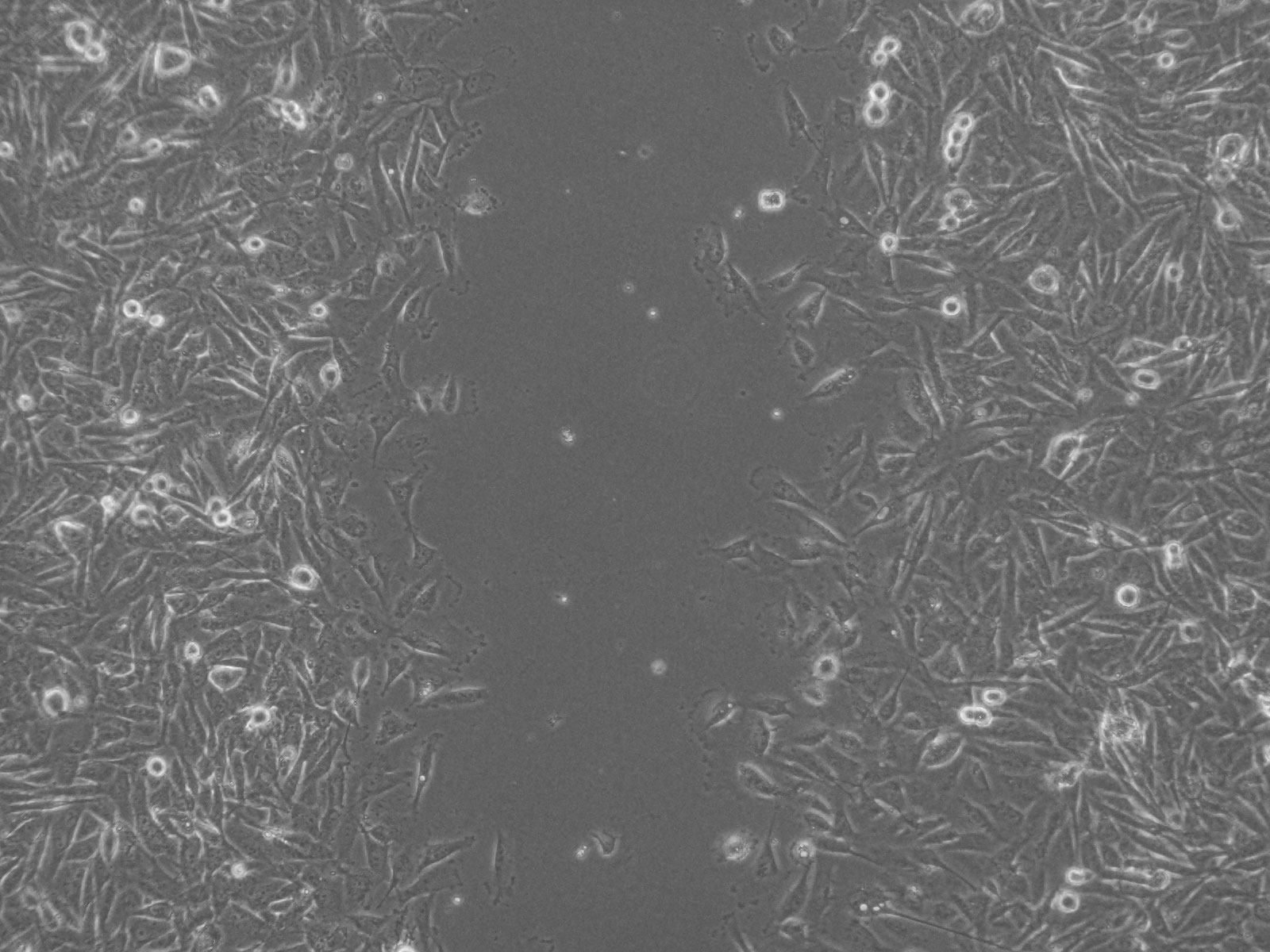

Supplement: Supplemental Information 8 [file peerj-10-14552-s008.zip › 图压缩版/Figure 4/U251-6H-NC.jpg]

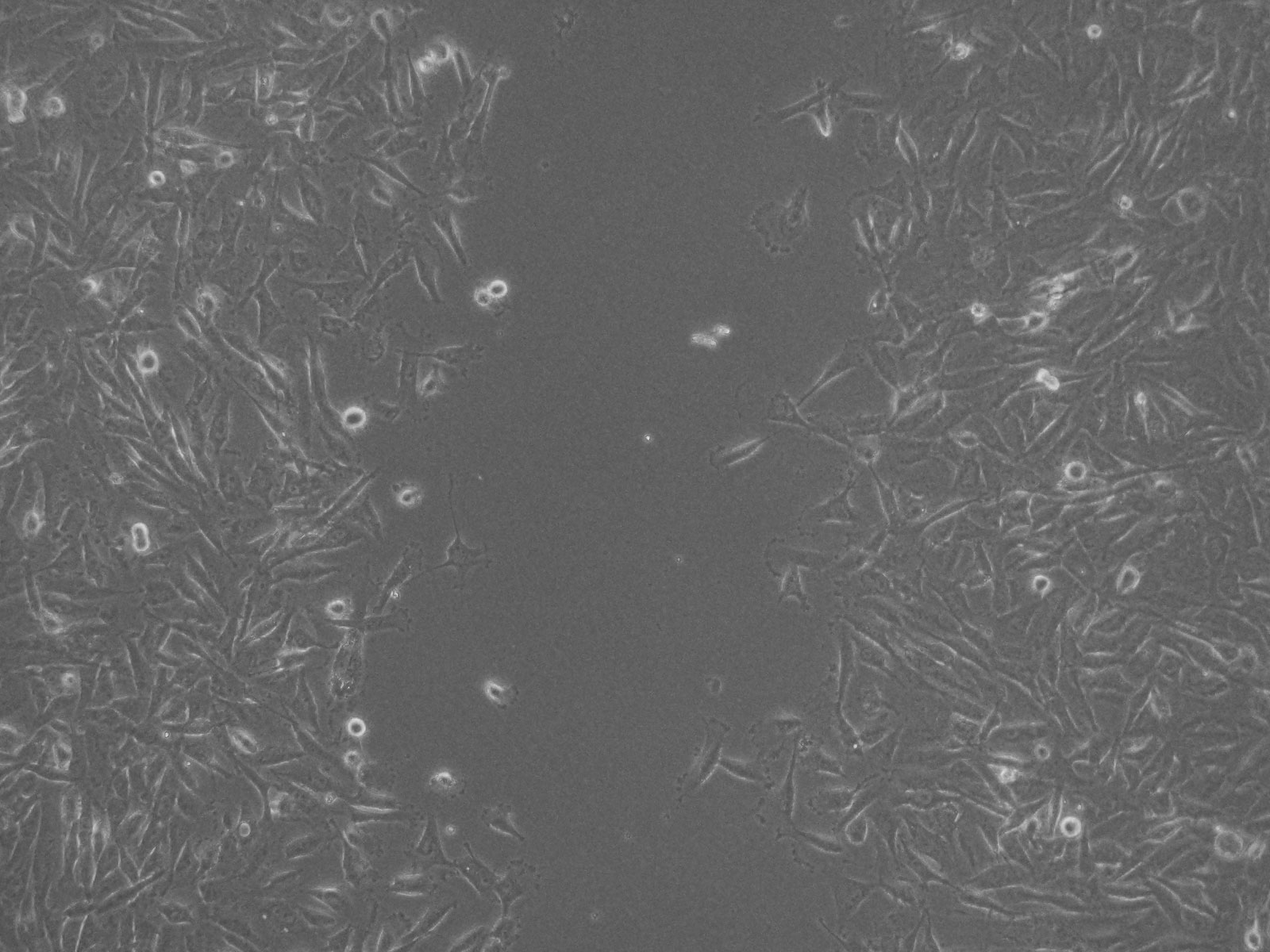

Supplement: Supplemental Information 8 [file peerj-10-14552-s008.zip › 图压缩版/Figure 4/U251-6H-s2-hsa_circ_008922.jpg]

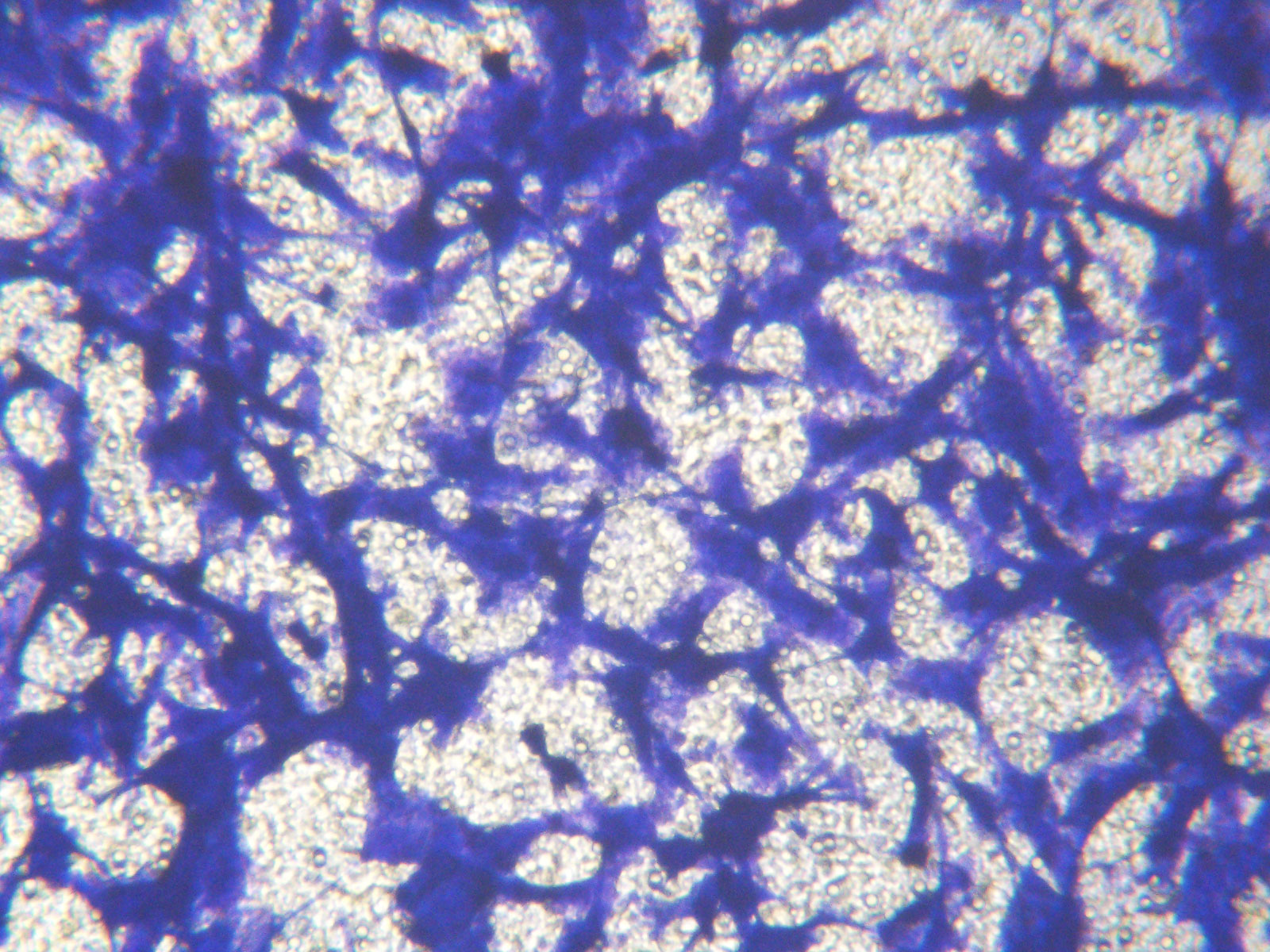

Supplement: Supplemental Information 8 [file peerj-10-14552-s008.zip › 图压缩版/Figure 5/A172-NC-Invasion.jpg]

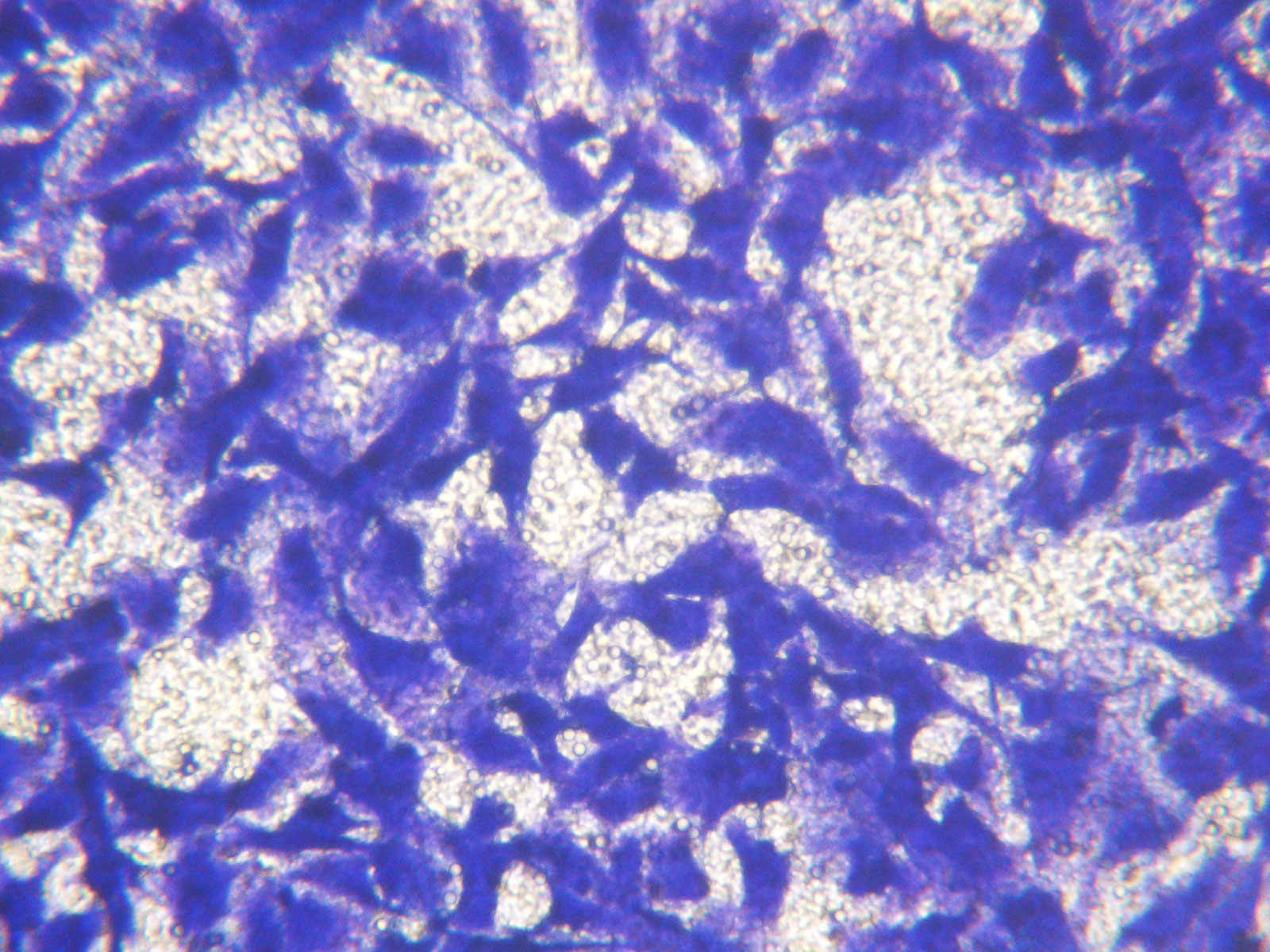

Supplement: Supplemental Information 8 [file peerj-10-14552-s008.zip › 图压缩版/Figure 5/A172-NC-Migration.jpg]

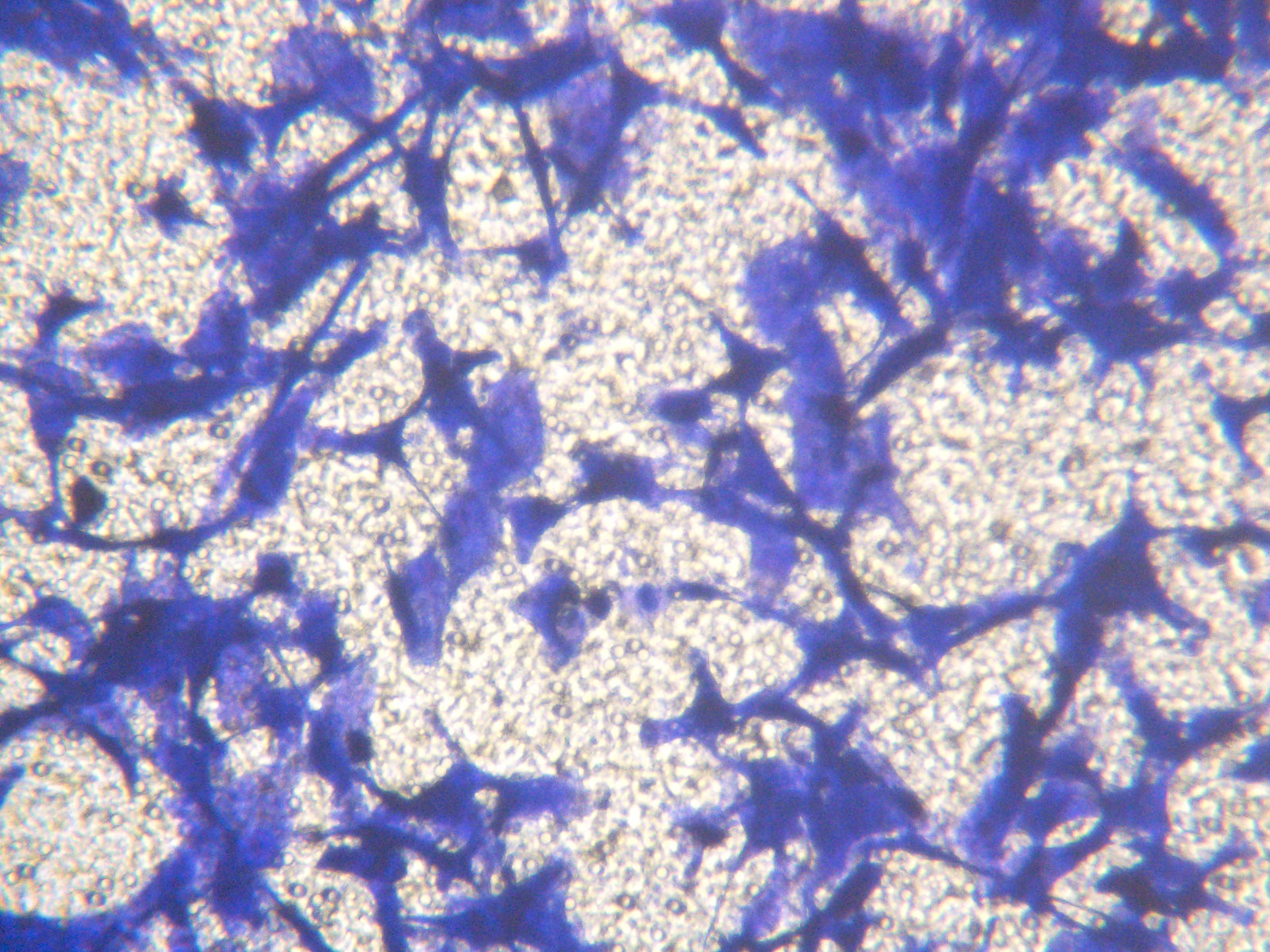

Supplement: Supplemental Information 8 [file peerj-10-14552-s008.zip › 图压缩版/Figure 5/A172-s2-hsa-circ0008922-Invasion.jpg]

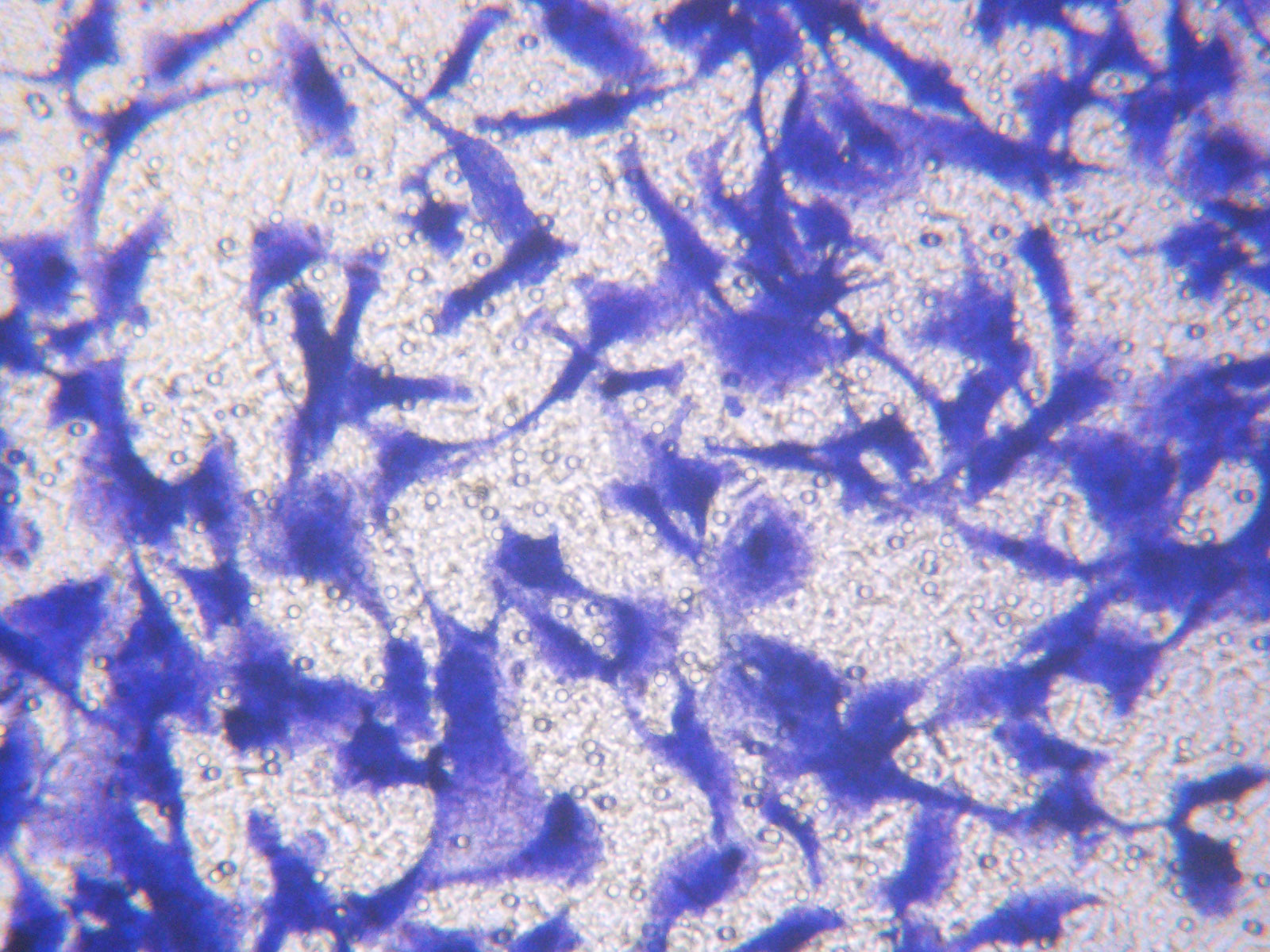

Supplement: Supplemental Information 8 [file peerj-10-14552-s008.zip › 图压缩版/Figure 5/A172-s2-hsa-circ0008922-Migration.jpg]

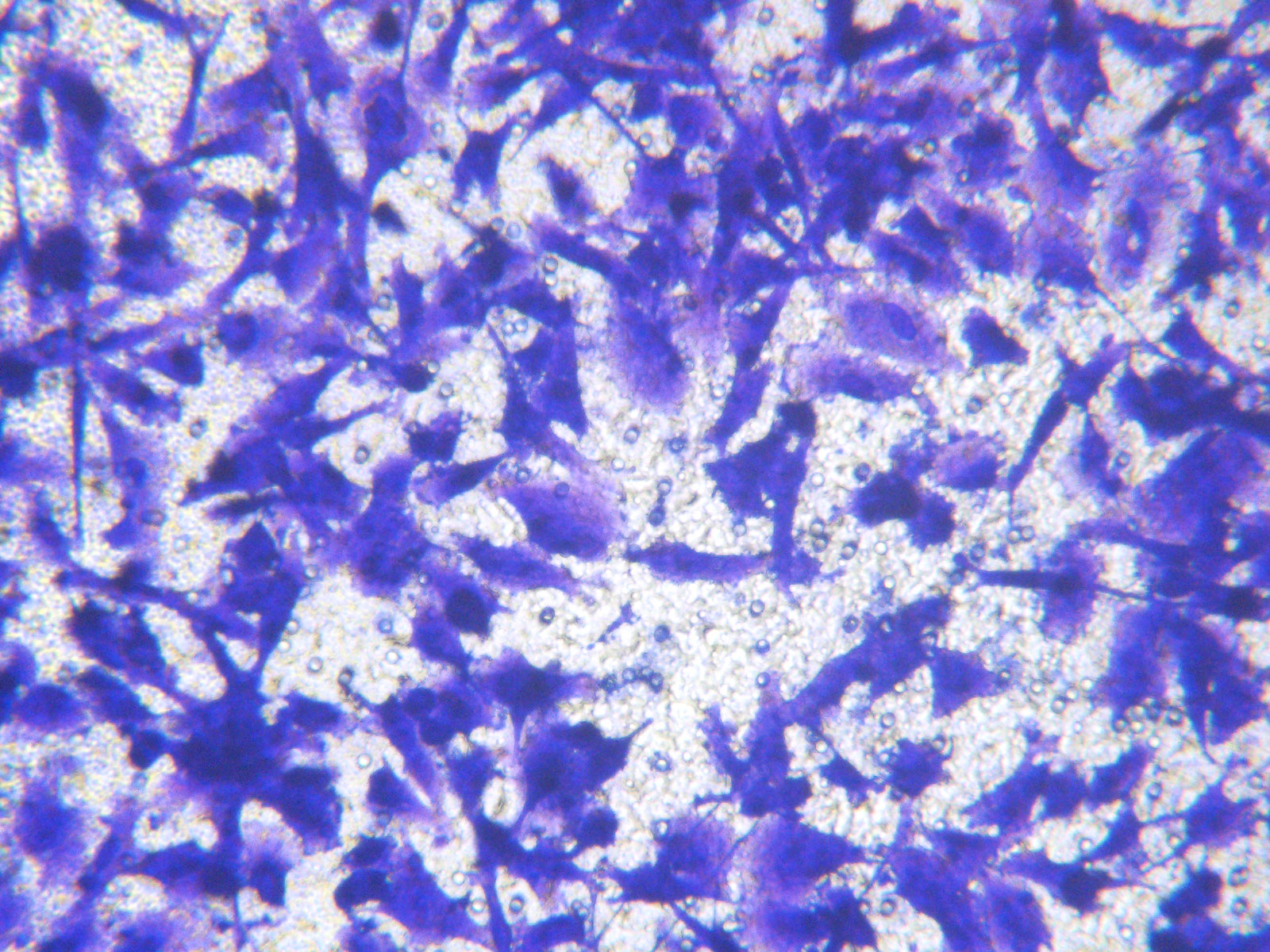

Supplement: Supplemental Information 8 [file peerj-10-14552-s008.zip › 图压缩版/Figure 5/U251-NC-Invasion.jpg]

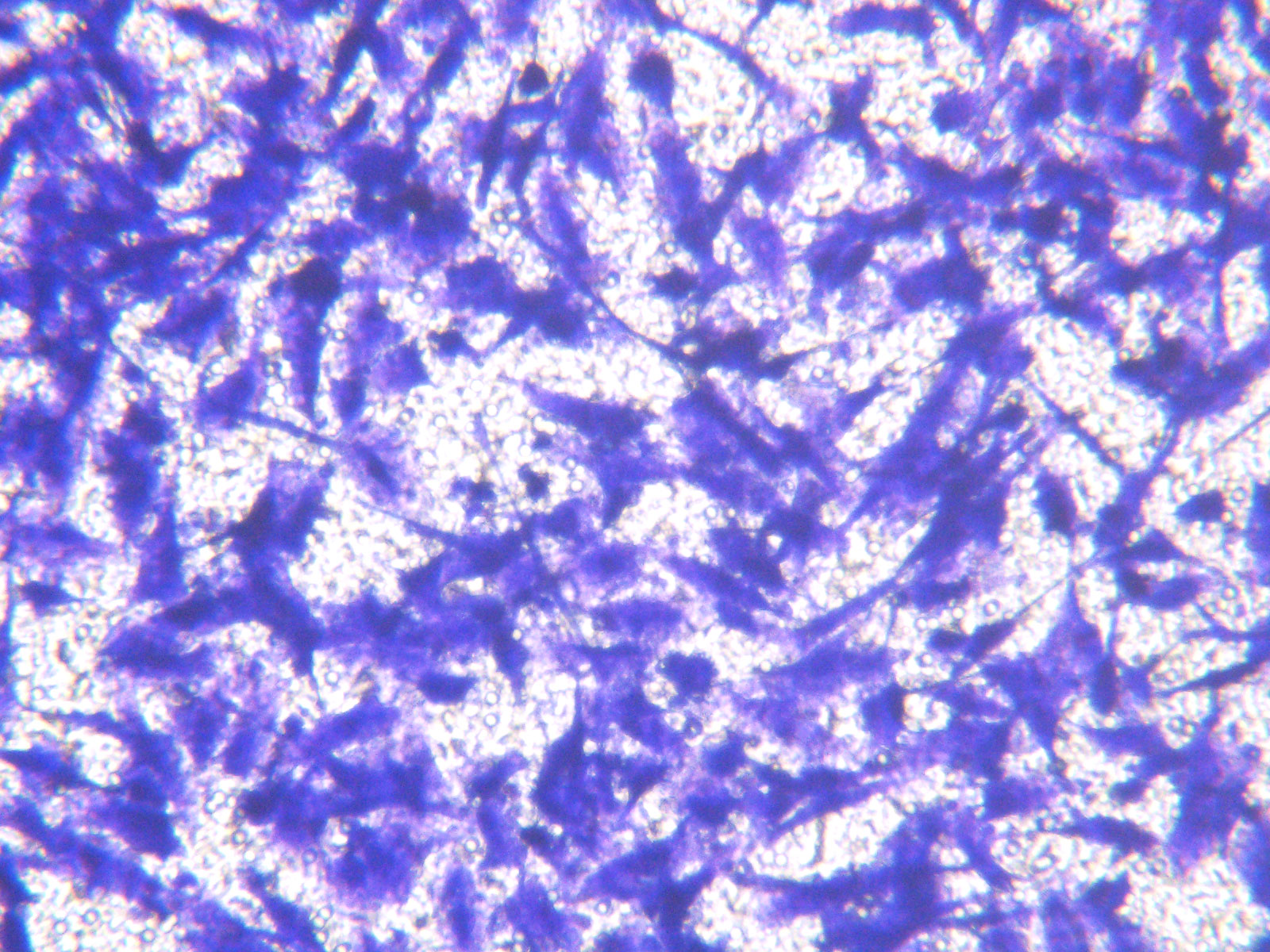

Supplement: Supplemental Information 8 [file peerj-10-14552-s008.zip › 图压缩版/Figure 5/U251-NC-Migration.jpg]

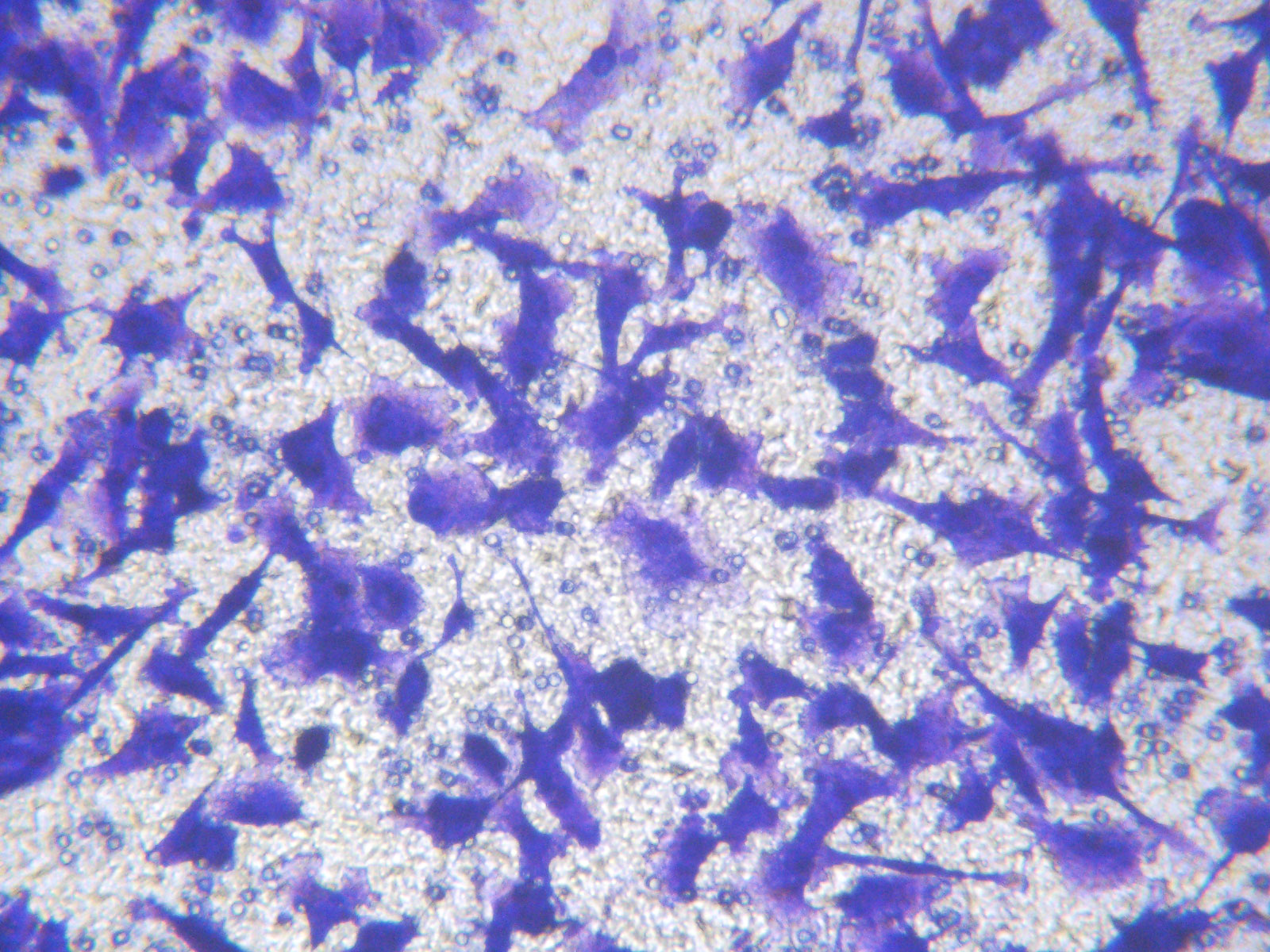

Supplement: Supplemental Information 8 [file peerj-10-14552-s008.zip › 图压缩版/Figure 5/U251-s2-hsa-circ0008922-Invasion.jpg]

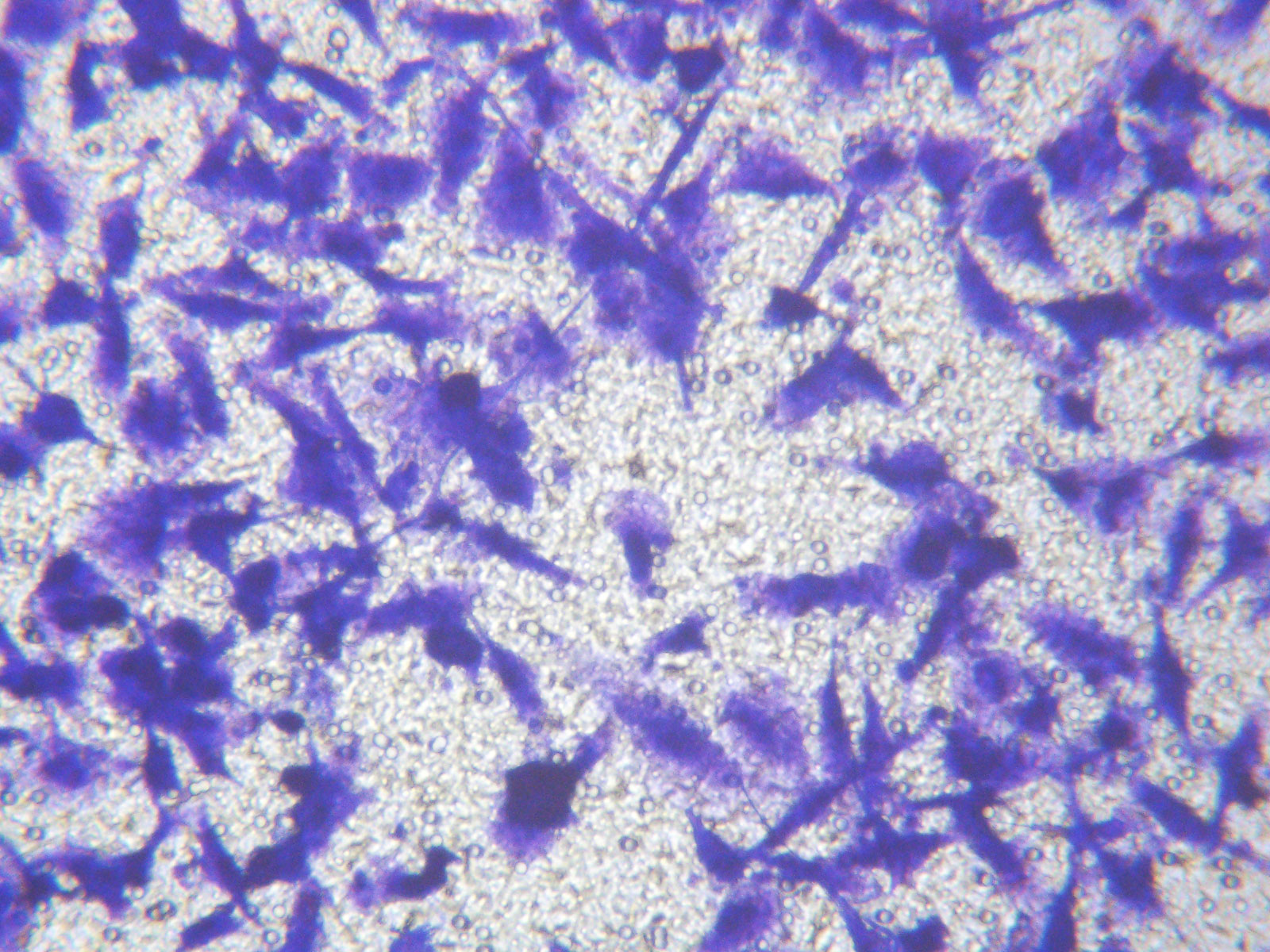

Supplement: Supplemental Information 8 [file peerj-10-14552-s008.zip › 图压缩版/Figure 5/U251-s2-hsa-circ0008922-Migration.jpg]

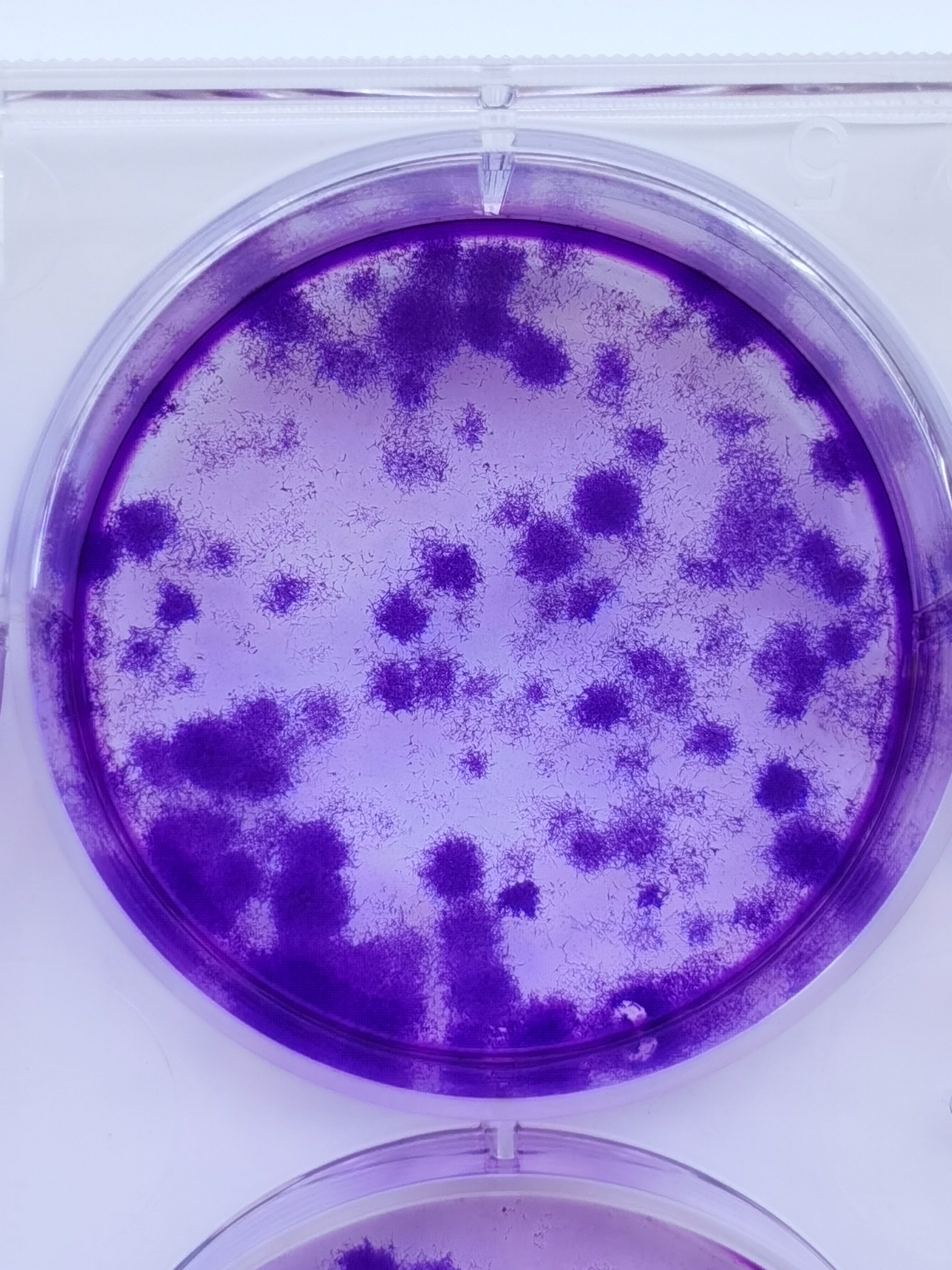

Supplement: Supplemental Information 8 [file peerj-10-14552-s008.zip › 图压缩版/Figure 6/A172 NC.jpg]

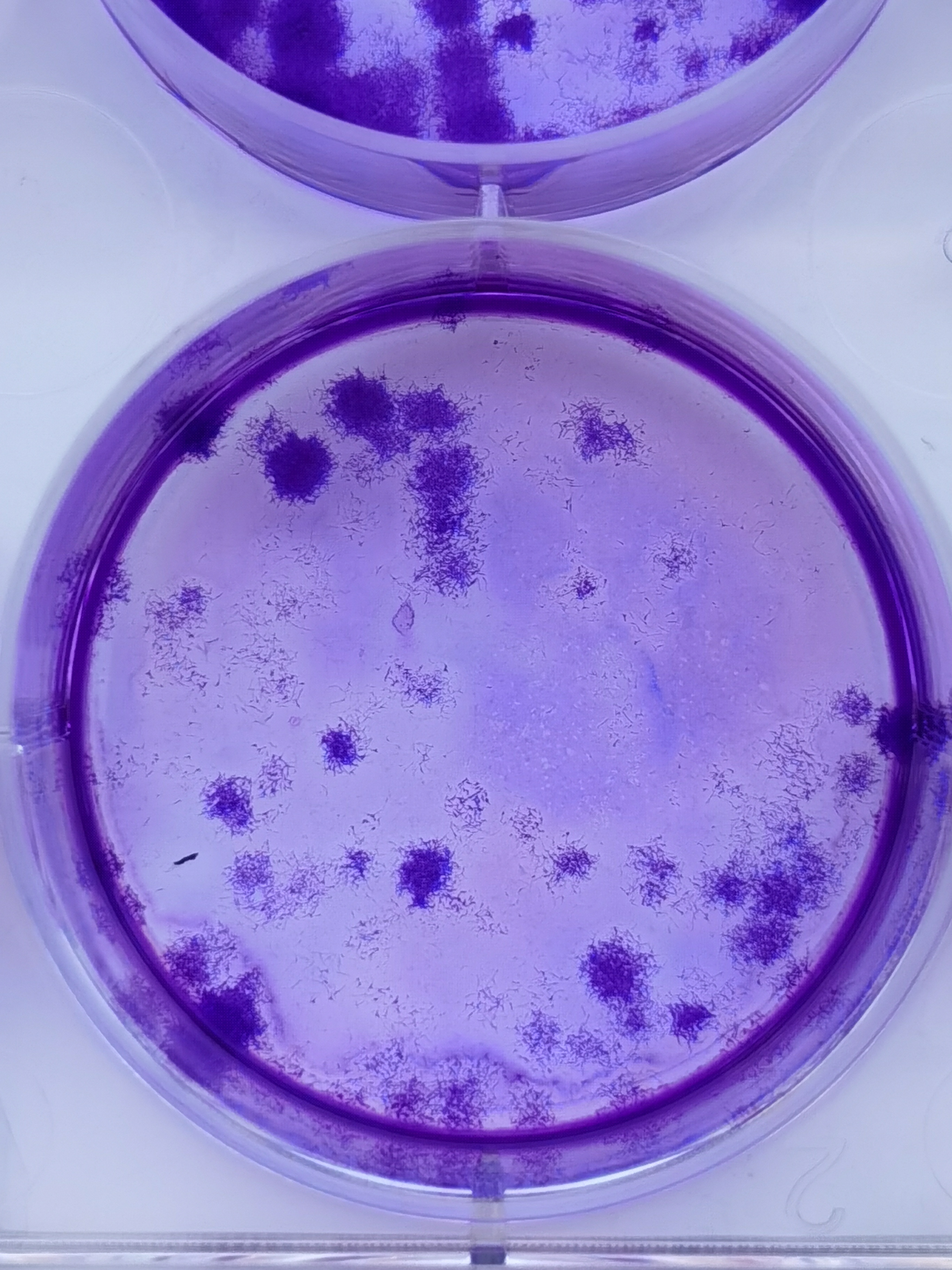

Supplement: Supplemental Information 8 [file peerj-10-14552-s008.zip › 图压缩版/Figure 6/A172 s2-hsa_circ-0008922.jpg]

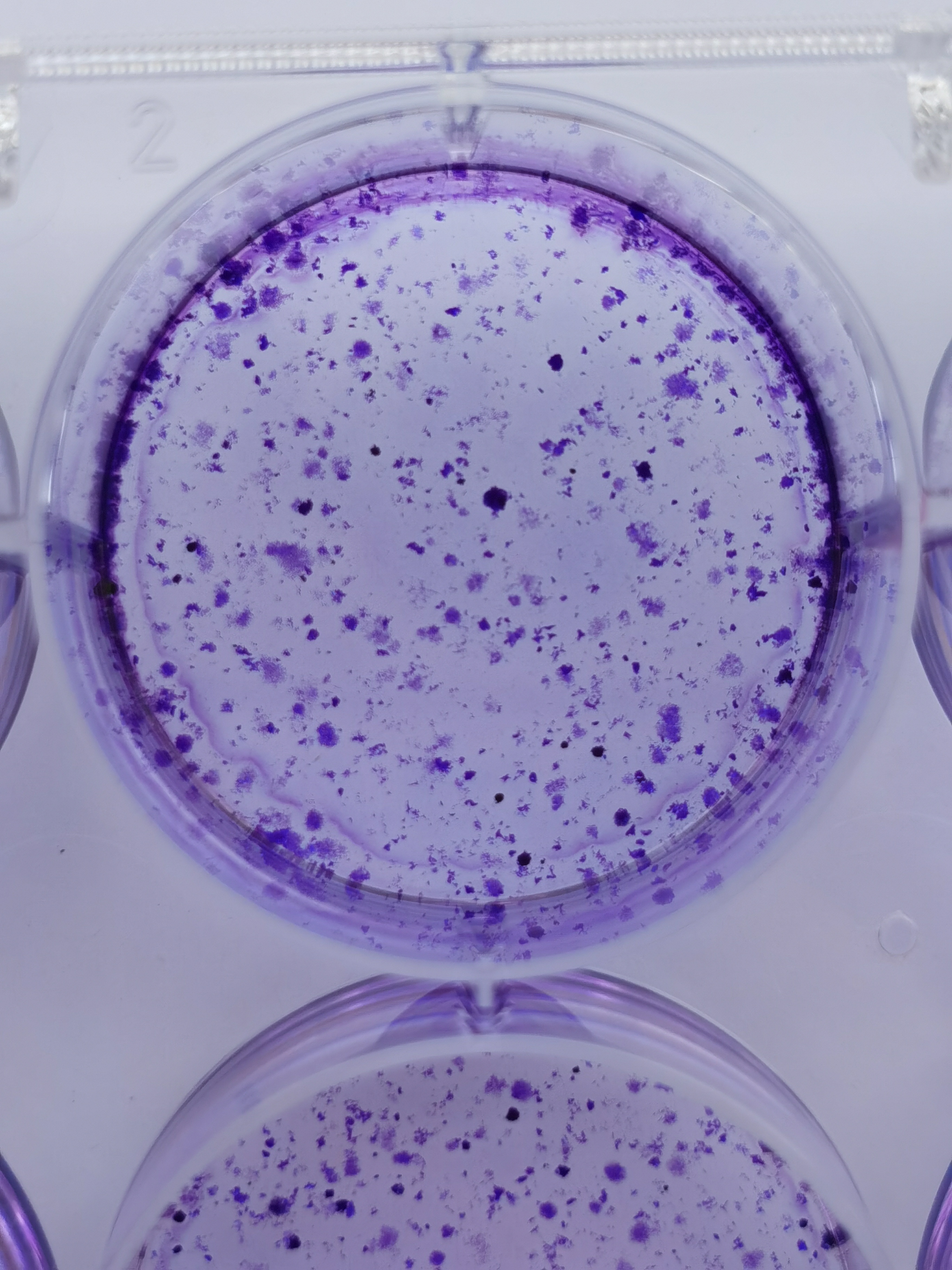

Supplement: Supplemental Information 8 [file peerj-10-14552-s008.zip › 图压缩版/Figure 6/U251 NC.jpg]

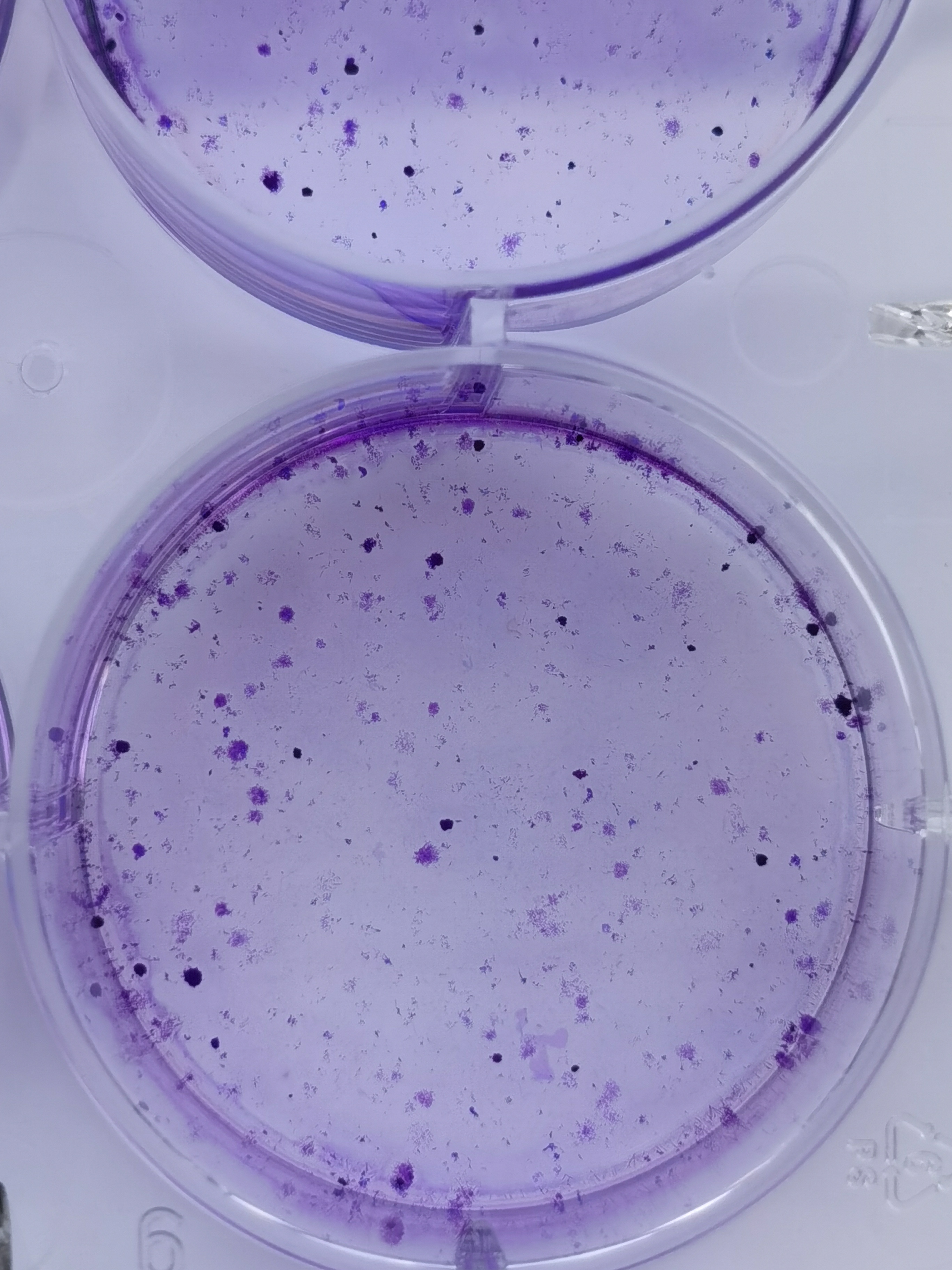

Supplement: Supplemental Information 8 [file peerj-10-14552-s008.zip › 图压缩版/Figure 6/U251 s2-hsa_circ-0008922.jpg]

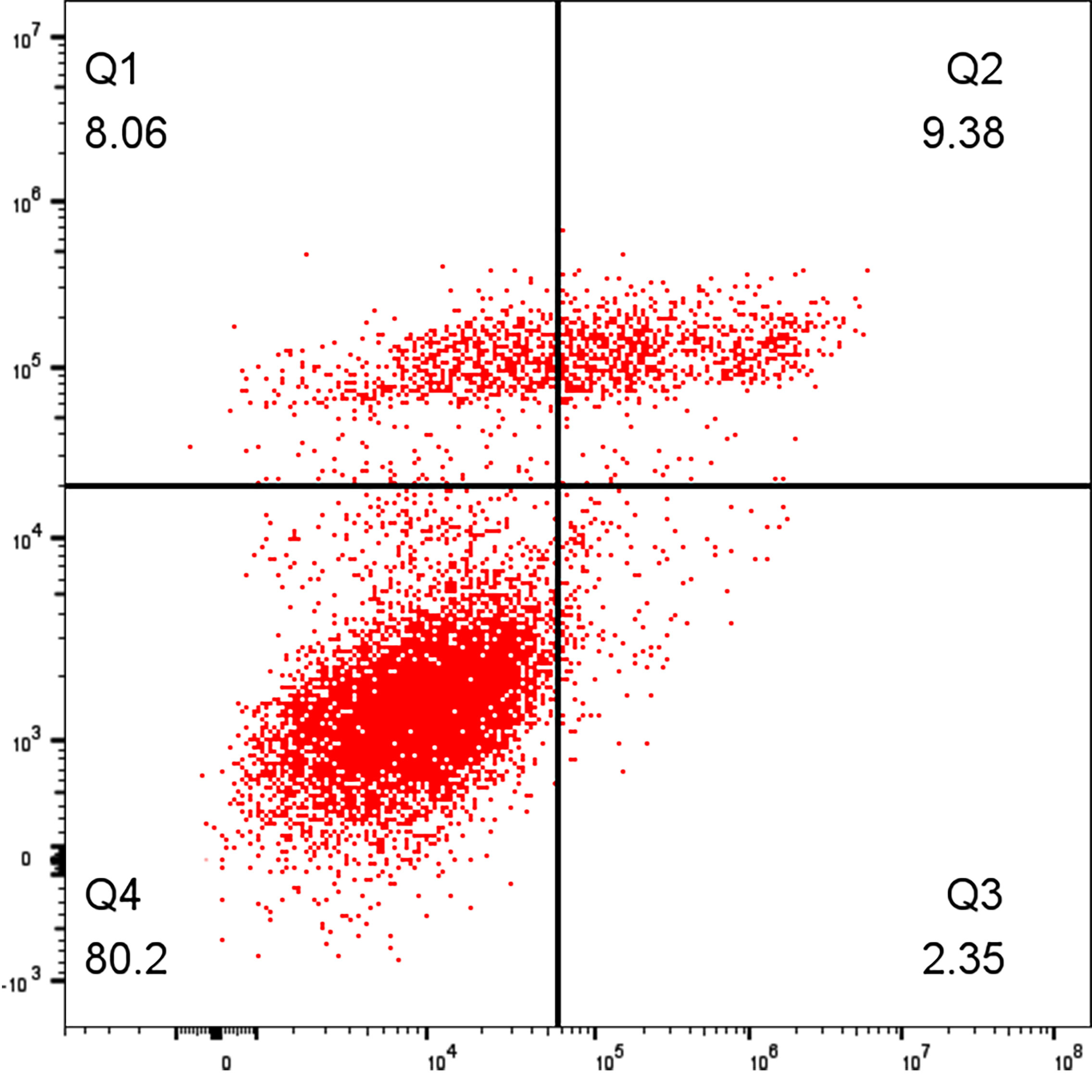

Supplement: Supplemental Information 8 [file peerj-10-14552-s008.zip › 图压缩版/Figure 7/A172-NC.jpg]

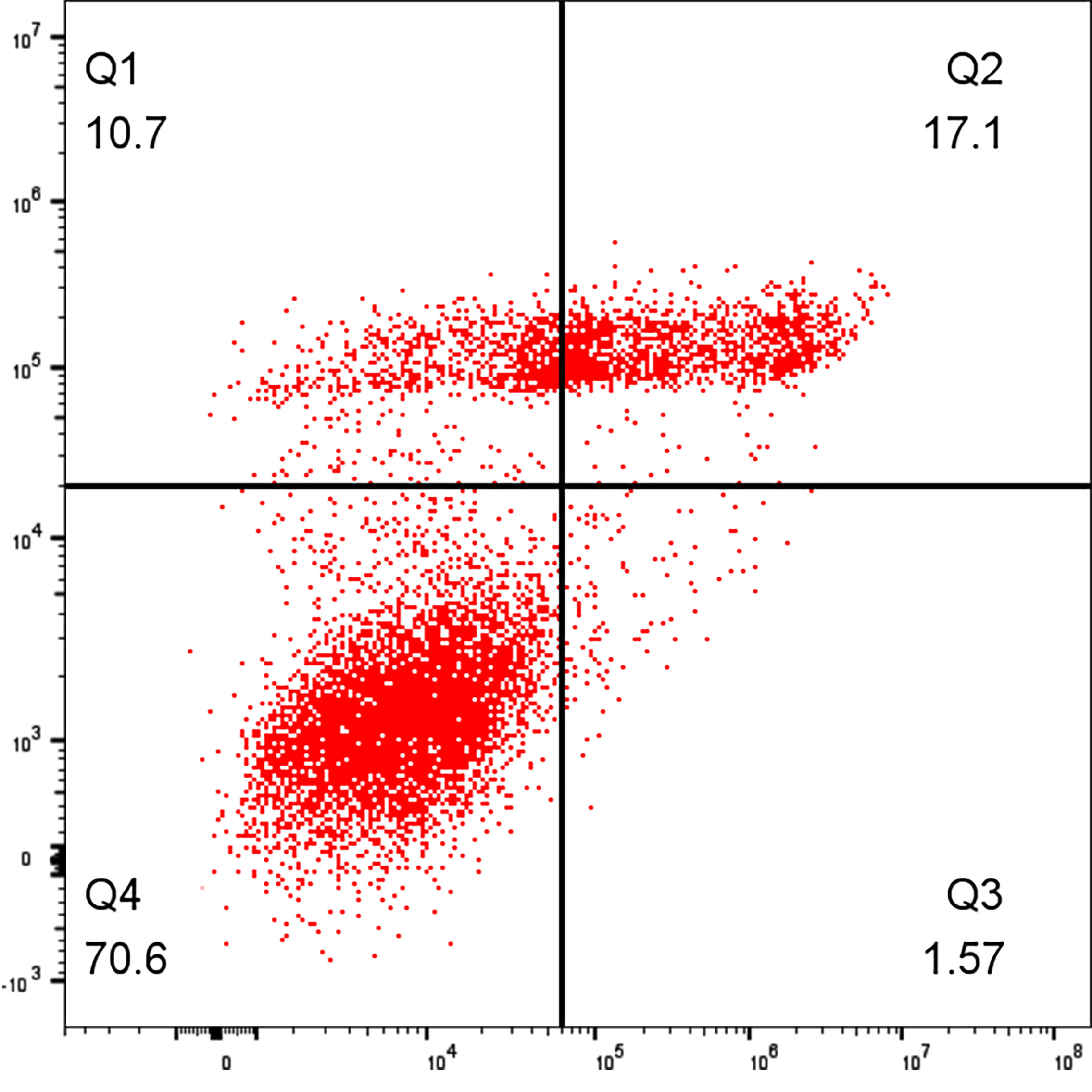

Supplement: Supplemental Information 8 [file peerj-10-14552-s008.zip › 图压缩版/Figure 7/A172-s2-hsa_circ-0008922.jpg]

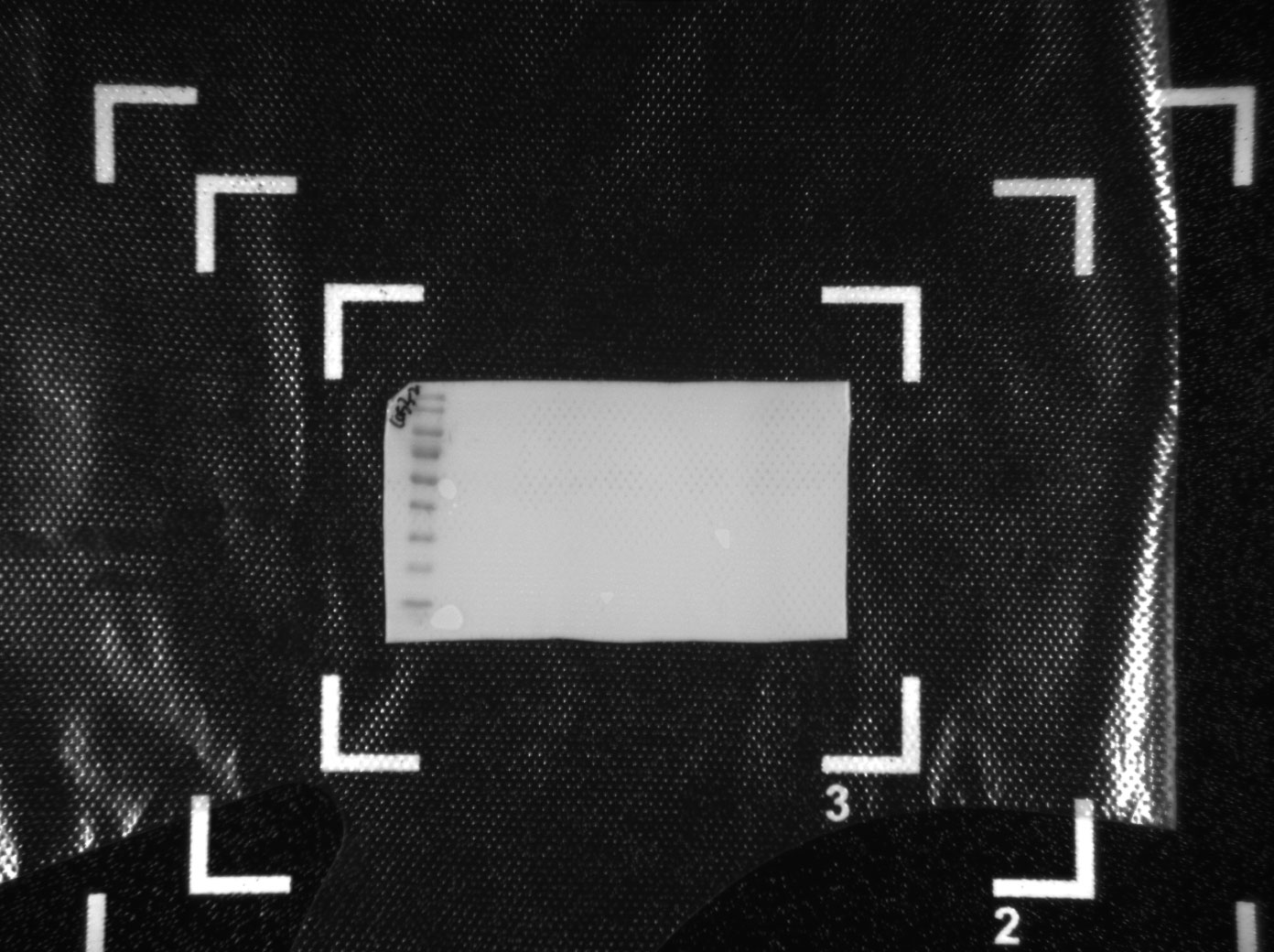

Supplement: Supplemental Information 8 [file peerj-10-14552-s008.zip › 图压缩版/Figure 7/CAS3-GA-Marker.jpg]

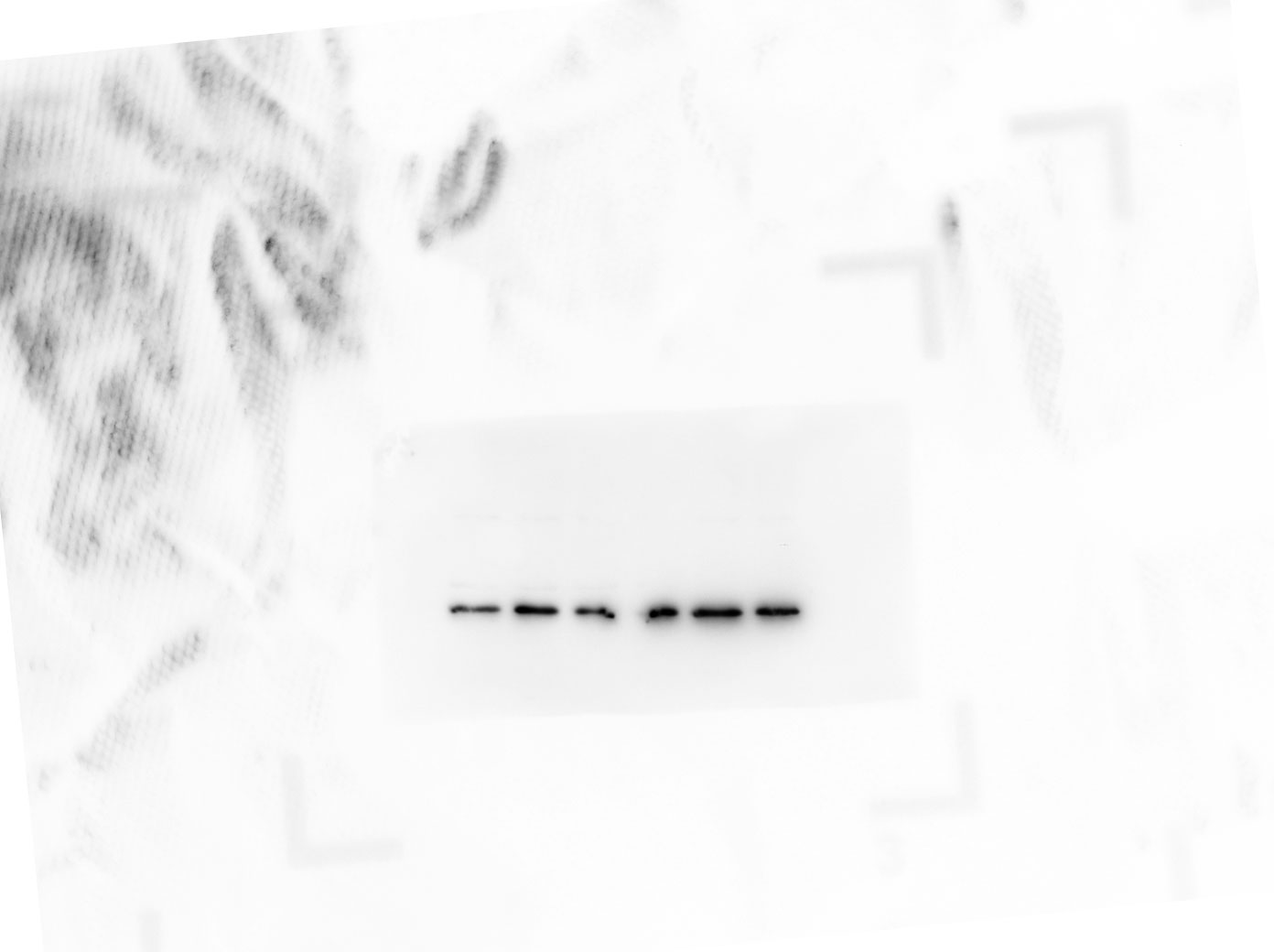

Supplement: Supplemental Information 8 [file peerj-10-14552-s008.zip › 图压缩版/Figure 7/CAS3.jpg]

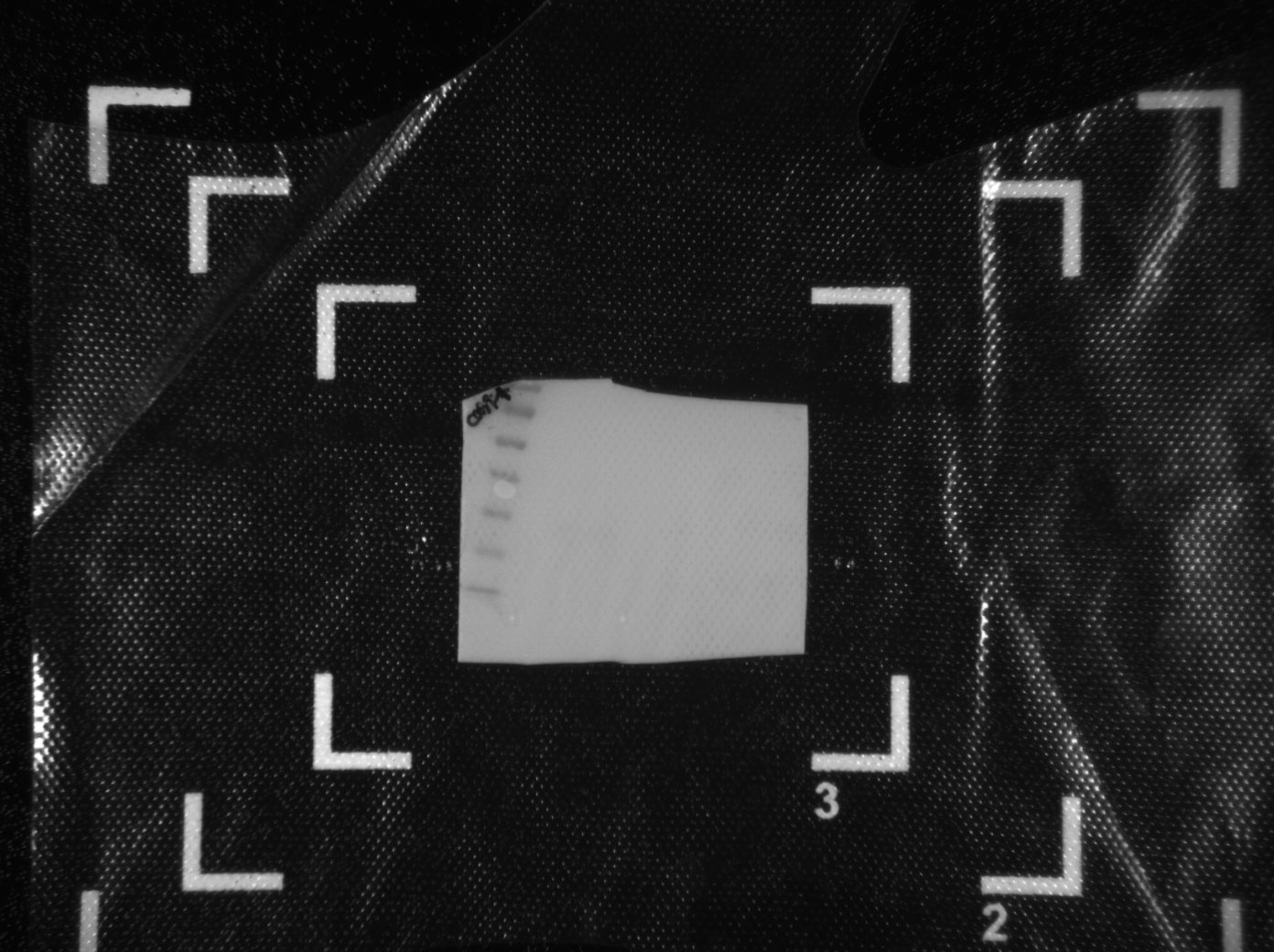

Supplement: Supplemental Information 8 [file peerj-10-14552-s008.zip › 图压缩版/Figure 7/CAS9-1-Marker-7.5.jpg]

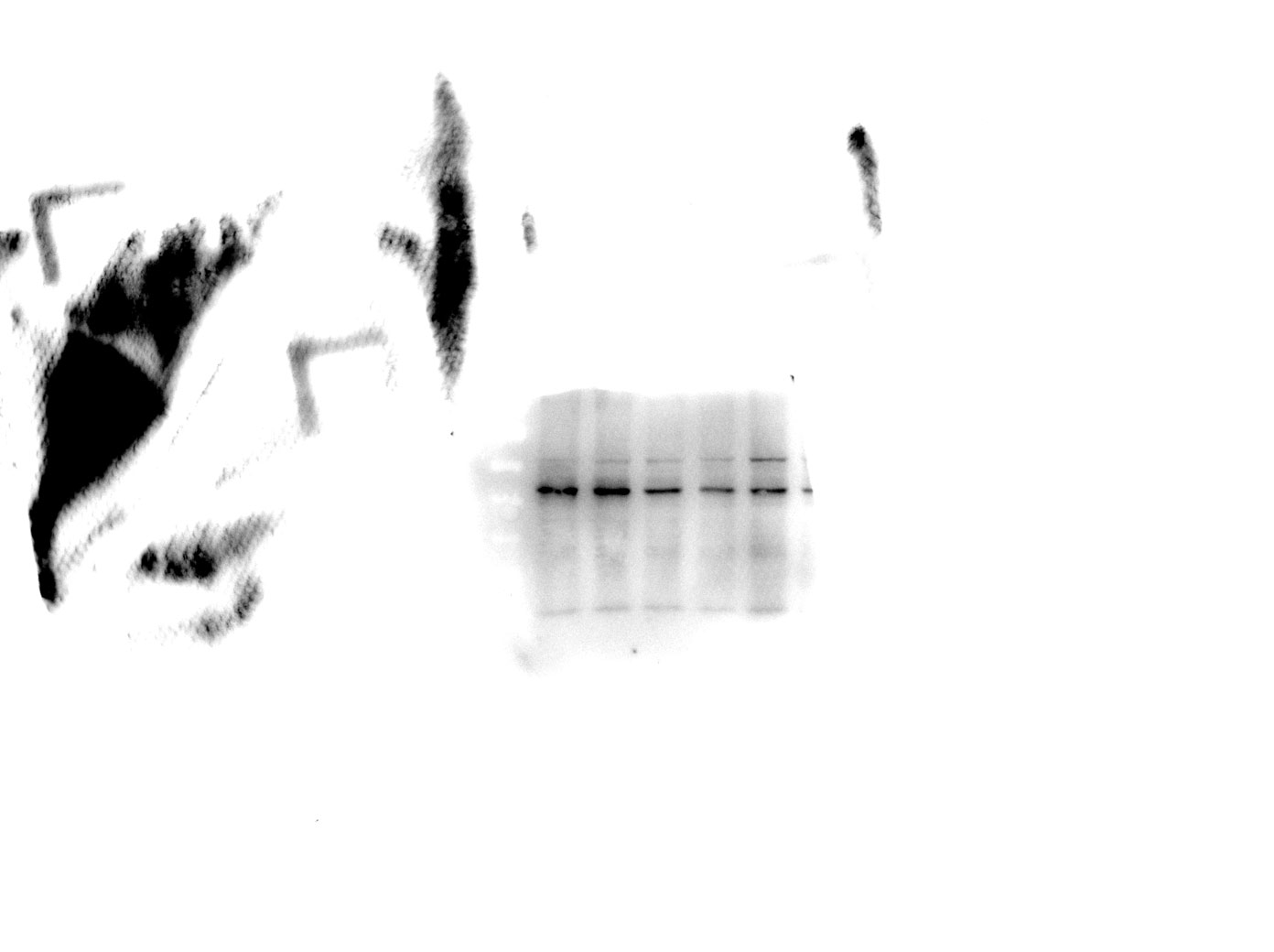

Supplement: Supplemental Information 8 [file peerj-10-14552-s008.zip › 图压缩版/Figure 7/CAS9.jpg]

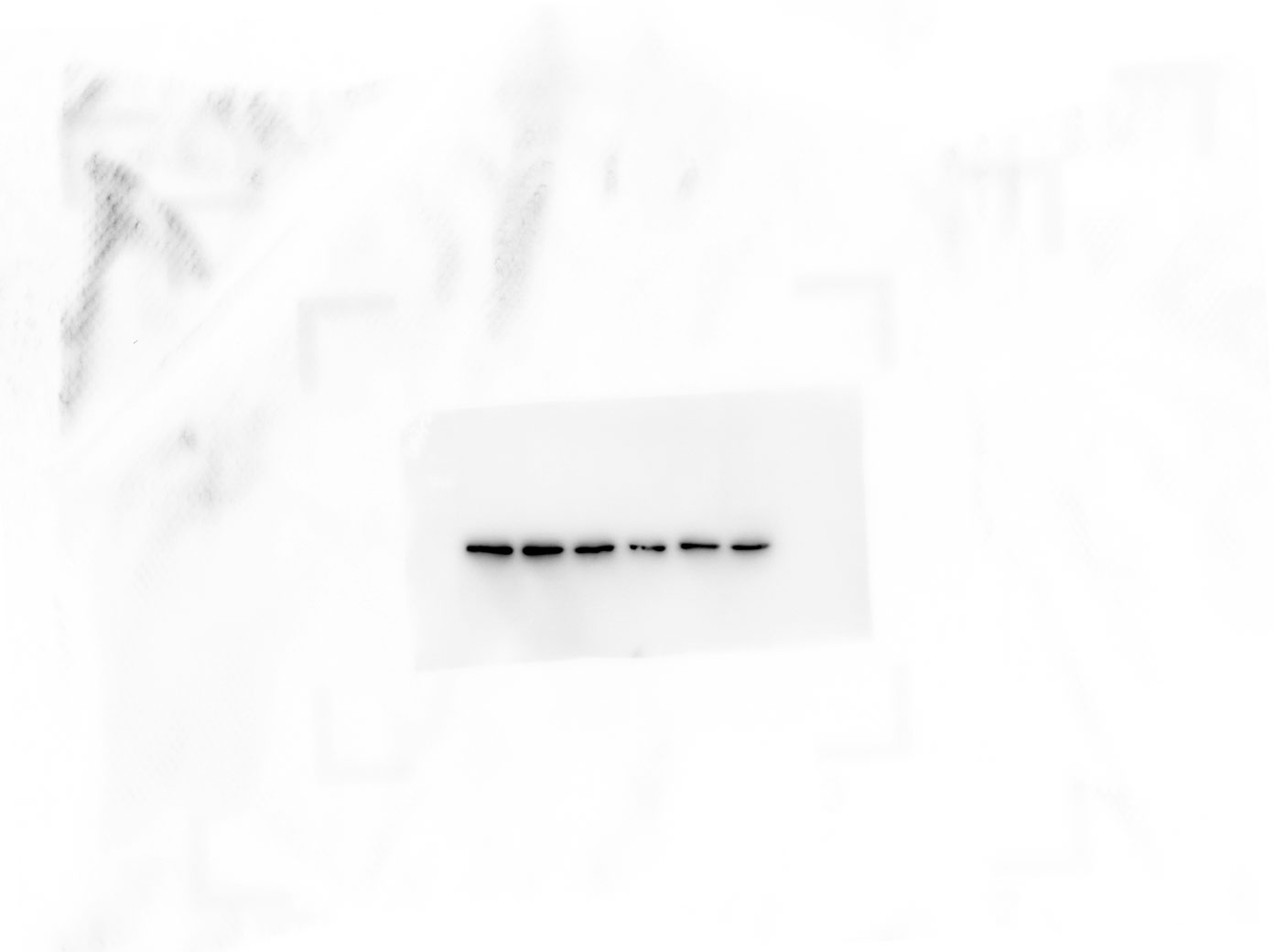

Supplement: Supplemental Information 8 [file peerj-10-14552-s008.zip › 图压缩版/Figure 7/GA-of-cas3.jpg]

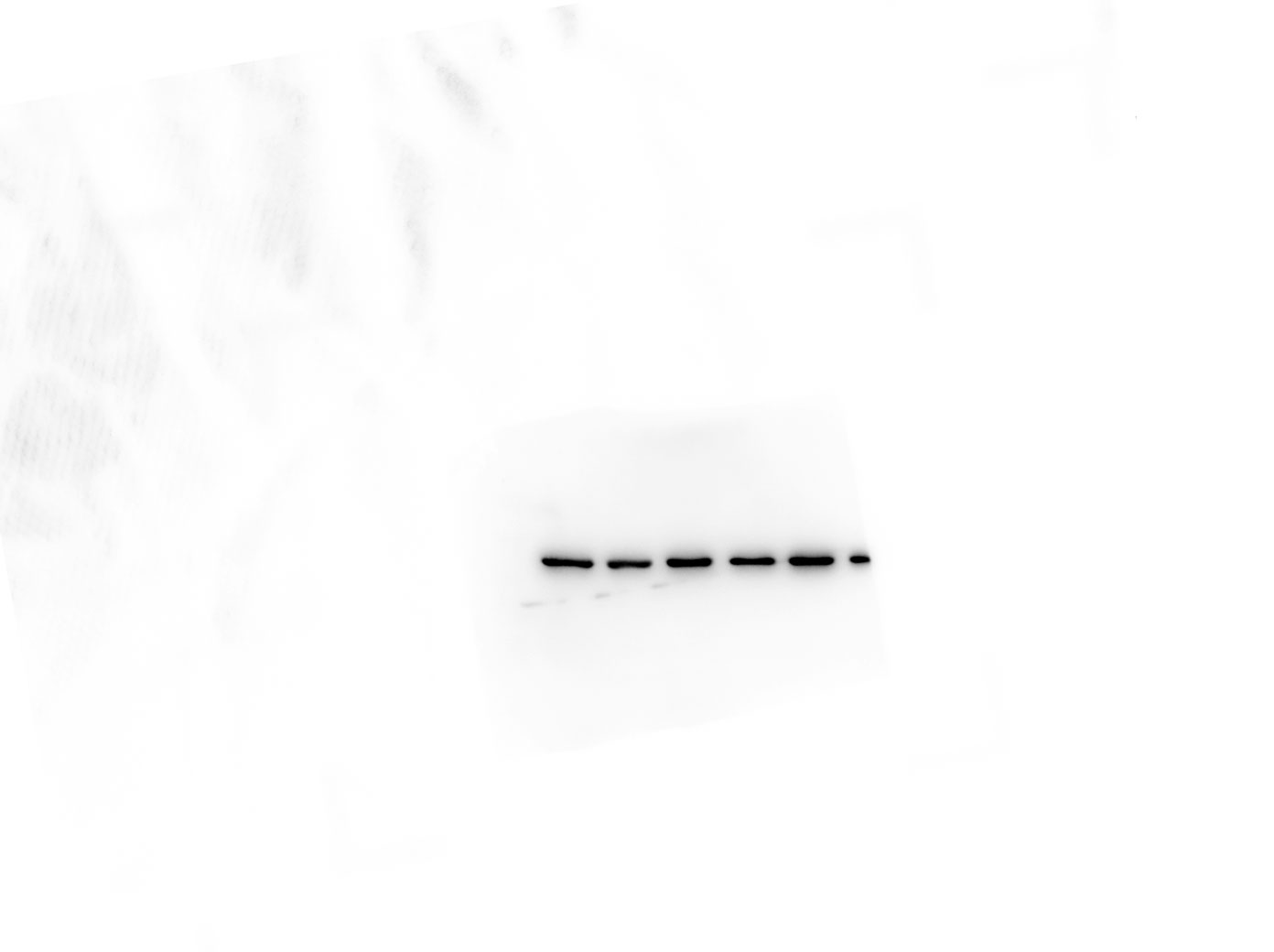

Supplement: Supplemental Information 8 [file peerj-10-14552-s008.zip › 图压缩版/Figure 7/GA-of-cas9.jpg]

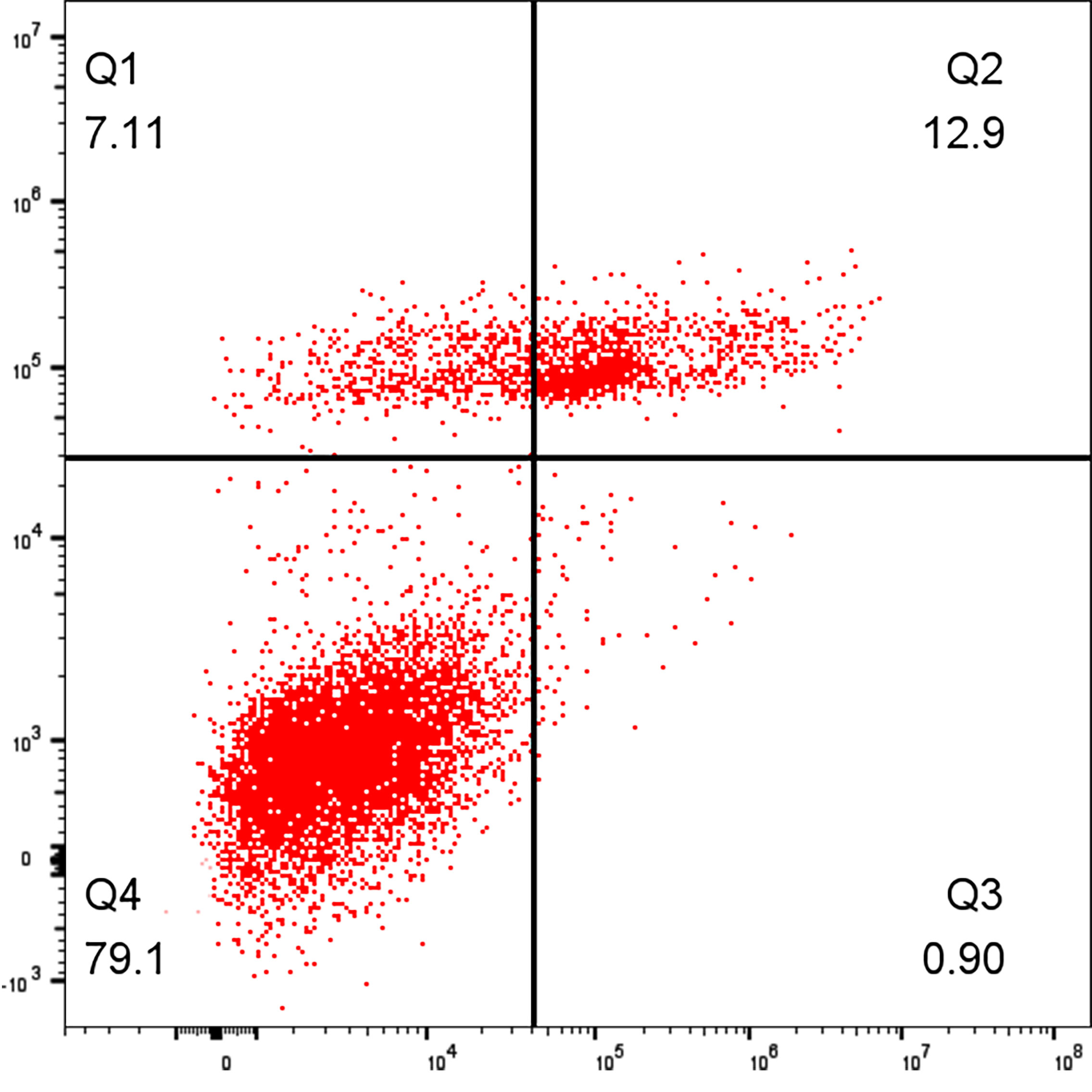

Supplement: Supplemental Information 8 [file peerj-10-14552-s008.zip › 图压缩版/Figure 7/U251-NC.jpg]

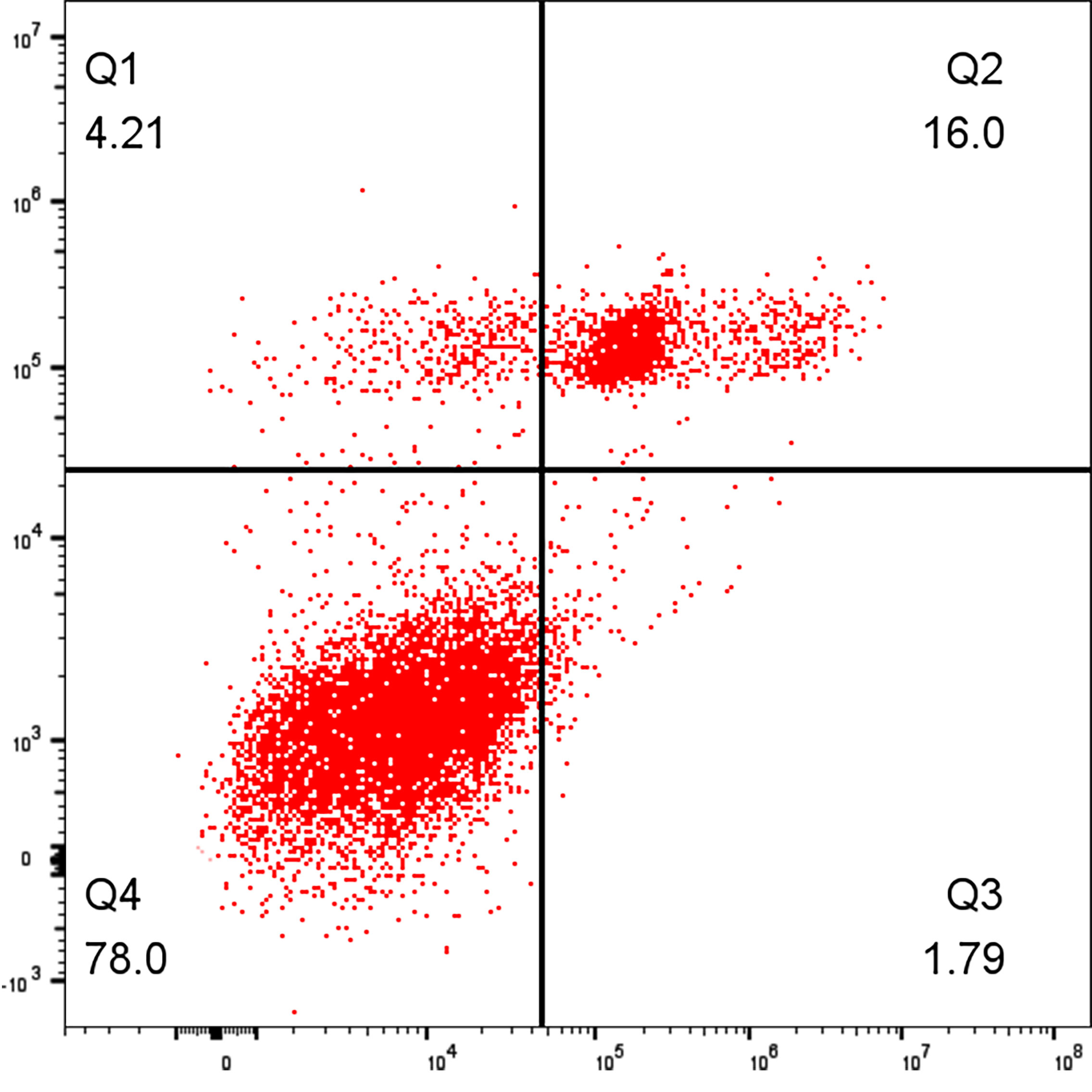

Supplement: Supplemental Information 8 [file peerj-10-14552-s008.zip › 图压缩版/Figure 7/U251-s2-hsa_circ-0008922.jpg]

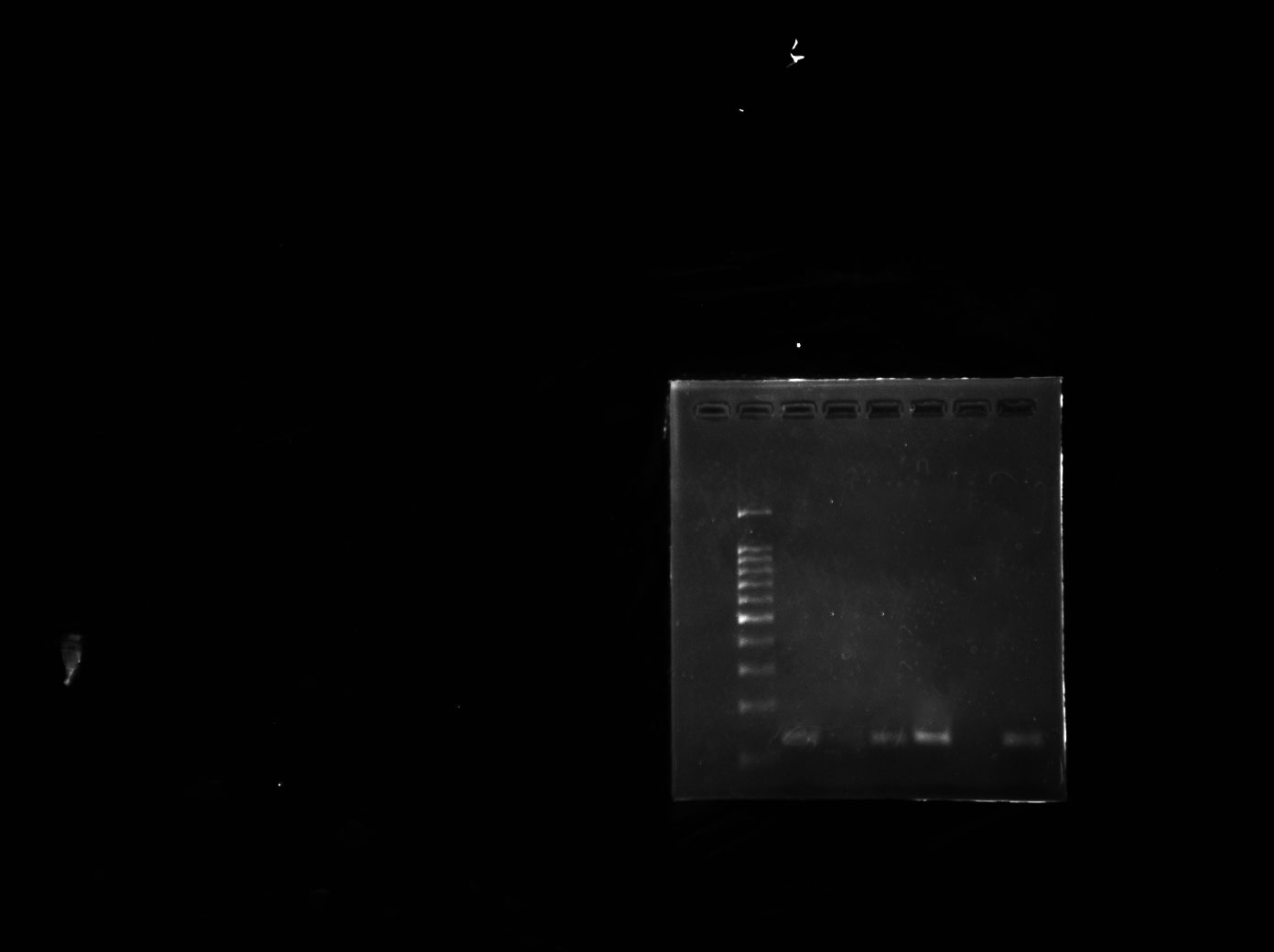

Supplement: Supplemental Information 8 [file peerj-10-14552-s008.zip › 图压缩版/Figure1.jpg]
